# AI-Enhanced Hybrid QAM–PPM Visible Light Communication for Body Area Networks

**DOI:** 10.3390/s26030971

**Published:** 2026-02-02

**Authors:** Shreyash Shrestha, Attaphongse Taparugssanagorn, Stefano Caputo, Lorenzo Mucchi

**Affiliations:** 1 Department of ICT, School of Engineering and Technology, Asian Institute of Technology, Klong Luang 12120, Thailand; shreyashshrestha22@gmail.com (S.S.); attaphongset@ait.asia (A.T.); 2 Department of Information Engineering, University of Florence, 50139 Florence, Italy; stefano.caputo@unifi.it

**Keywords:** CNN–transformer, deep learning, hybrid modulation, optical wireless communication, visible light communication, body area networks

## Abstract

This paper investigates an artificial intelligence (AI)-enhanced visible light communication (VLC) system for body area networks (BANs) based on a hybrid modulation framework that jointly employs quadrature amplitude modulation (QAM) and pulse-position modulation (PPM). The dual-modulation strategy leverages the high spectral efficiency of QAM together with the robustness of PPM to light-emitting diode (LED) nonlinearity and timing distortions, enabling simultaneous high-rate and reliable communication, two essential requirements in BAN applications. To address the nonlinear response of light-emitting diodes and the variability in indoor optical channels, the system integrates classical predistortion techniques with a deep learning equalizer combining convolutional neural network (CNN)–transformer layers. This hybrid model captures both local and long-range distortion patterns, improving symbol reconstruction for both modulation branches. The study further examines pilot-assisted equalization and adaptive bit loading, showing that these strategies strengthen link robustness under diverse channel conditions while enhancing spectral efficiency. The proposed architecture demonstrates that combining dual modulation with AI-driven equalization and adaptive transmission strategies leads to a more resilient and efficient VLC system, well-suited for the dynamic constraints of wearable and body-centric communication environments.

## 1. Introduction

Visible light communication (VLC) has emerged as a viable short-range wireless technology for dense and interference-sensitive environments, particularly in applications where radio frequency (RF) systems face limitations [1]. Its use of intensity-modulated LED sources enables high data rates and reduced signal leakage outside the illuminated area, making it attractive for wearable and body-centric communications. However, practical VLC systems are still constrained by the nonlinear electro-optical characteristics of LEDs, multipath reflections, and ambient light fluctuations. These impairments distort the transmitted waveform, degrade symbol decision boundaries, and limit the achievable spectral efficiency, especially when high-order modulation is required. Recent advances in artificial intelligence have shown promise in modeling and compensating such nonlinear effects, offering more flexible and data-driven alternatives to traditional equalizers (see Section 1.1). Building on this trend, this work explores a hybrid modulation- and deep learning-based approach to improve the robustness and efficiency of VLC links in body area network scenarios.

The practical deployment of VLC systems is still constrained by several physical and device-level impairments that significantly affect link reliability. Indoor environments introduce complex multipath components due to reflections from walls, furniture, and moving bodies, causing temporal dispersion and inter-symbol interference (ISI). Ambient illumination from sunlight, fluorescent lamps, or LED luminaires adds unpredictable shot noise that further deteriorates signal quality. In addition, the nonlinear electro-optical response and limited modulation bandwidth of commercial LEDs distort high-order and high-rate waveforms, making equalization increasingly challenging. User mobility and dynamic occlusions introduce rapid channel fluctuations that conventional linear compensation methods struggle to track. Collectively, these effects reduce the achievable spectral efficiency and substantially degrade bit error rate (BER) performance, particularly in scenarios requiring compact low-power devices such as body area networks [2].

Hybrid modulation schemes, such as combining pulse-position modulation (PPM) with high-order constellations like quadrature phase shift keying (QPSK), have shown that exploiting complementary signaling formats can improve link reliability, particularly when supported by neural network-based detectors [3]. Using multiple modulation types enables VLC systems to flexibly operate across low- and high-data-rate regimes, increasing robustness to channel variability and practical impairments. Machine learning-assisted intensity modulation/direct detection (IM/DD) techniques further enhance performance by learning nonlinear distortion patterns that conventional equalizers cannot fully capture. Prior studies also demonstrated that deep learning models, including long short-term memory (LSTM) networks combined with maximum likelihood detection under accurate channel state information (CSI), can effectively model LED nonlinearities and outperform classical signal processing approaches [4].

This study proposes a hybrid VLC framework that combines dual-modulation signaling with advanced deep learning-based equalization to improve link reliability under practical channel impairments. A convolutional neural network (CNN)–transformer network (TFN) architecture is introduced to jointly address LED nonlinearities and distortion patterns that conventional receivers cannot fully compensate. In addition, the system compares classical polynomial predistortion with a data-driven CNN-based predistorter, demonstrating the advantages of learned nonlinear compensation. Adaptive bit loading (ABL) is further integrated to adjust modulation order according to instantaneous channel conditions, enhancing spectral efficiency under varying signal-to-noise ratio (SNR) levels. Together, these strategies enable a more robust and flexible end-to-end VLC system that better reflects real-world operating scenarios.

Body area networks (BANs) impose stricter constraints than typical indoor VLC links, including short-range operation, strong body-shadowing effects, mobility-induced channel variations, and the need for high reliability at low power. The proposed combination of hybrid modulation, learned nonlinear compensation, and adaptive bit loading directly addresses these challenges by enhancing robustness under rapid channel fluctuations and improving efficiency within the limited optical power budgets typical of wearable devices. This makes the proposed framework particularly suited for BAN scenarios, where both reliability and adaptability are essential.

Despite the recent advances in visible light communication systems, several limitations remain that hinder reliable and efficient operation in practical scenarios. High-order modulation schemes, such as QAM-based OFDM, provide high spectral efficiency but are highly sensitive to LED nonlinearity and channel distortions. Conversely, more robust schemes such as PPM exhibit strong resilience to amplitude impairments but suffer from low spectral efficiency. In addition, many existing AI-assisted VLC receivers focus on compensating either channel impairments or hardware nonlinearity in isolation, without jointly addressing modulation adaptability and efficiency, particularly in body area network scenarios characterized by short-range operation and dynamic channel conditions.

The objective of this work is to address these limitations by proposing a unified VLC framework that combines complementary modulation formats with deep learning-based equalization and adaptive transmission. Specifically, the proposed approach integrates hybrid QAM–PPM modulation, a CNN–transformer equalizer for mitigating LED nonlinearity and channel impairments, and adaptive bit loading to improve spectral efficiency under varying link conditions. The framework is designed with body area network applications in mind, where robustness, adaptability, and efficient use of limited optical resources are critical.

### 1.1. Related Works

Visible light communication (VLC) has emerged as a critical technology to alleviate congestion in traditional radio frequency (RF) bands, which are increasingly strained due to the rapid growth of connected devices. By leveraging visible light, VLC provides a dual function of illumination and high-speed data transmission, offering an alternative to RF communication while enhancing overall system capacity [5]. VLC has shown significant promise in medical applications, where reliable data transfer is vital for patient monitoring and timely interventions, as well as in the Internet of Things (IoT) domain, supporting industrial automation and mobility systems that require robust low-latency communication [6].

A different application of VLC can be found in underwater optical wireless communication, as demonstrated in [7]. In their study, a laser diode (LD) was deployed in the Arabian Gulf, transmitting non-return-to-zero on–off keying (NRZ-OOK) signals over varying bit rates. The measurements were collected at 1 m intervals through the underwater channel. The LD operated at three different power levels: 24, 26, and 28 dBm. Their results showed that the convolutional neural network (CNN)-based signal recovery approach successfully mitigated channel impairments, achieving high signal fidelity and demonstrating strong potential for underwater VLC applications [7].

Artificial intelligence (AI) and machine learning (ML) techniques have been increasingly applied to VLC systems to improve signal detection, channel estimation, and overall reliability. Hybrid modulation schemes, such as OOK combined with orthogonal frequency division multiplexing (OFDM), allow VLC systems to efficiently handle both low- and high-data-rate symbols. Moreover, long short-term memory (LSTM)-based decoders significantly reduce bit error rate (BER) and improve system performance [8]. Deep learning models, including CNNs and LSTMs, have achieved performance close to maximum likelihood detection even under limited channel state information (CSI), effectively capturing nonlinearities and distortions introduced by LEDs [4]. These AI-driven enhancements enable VLC systems to adapt to dynamic indoor environments and varying channel conditions, making them increasingly viable for real-world deployment.

Recent works focus on enhancing VLC robustness through advanced deep learning-based dataset modeling. In particular, biomedical data transmission benefits from semantic communication and jamming techniques. Embedding a cryptographic key in each node enables semantic encoding, while jamming further strengthens communication security. These methods demonstrate substantial improvements in both reliability and security for AI-enhanced VLC systems [9]. In contrast, the present work does not address semantic security, jamming, or adversarial threats and focuses exclusively on physical-layer signal reconstruction under nonlinear and noisy VLC channels.

In their study, Qian et al. investigated the use of discrete multi-tone (DMT) modulation in nonlinear VLC systems. They demonstrated that the post-distortion (PD) algorithm significantly improves BER performance by distributing nonlinear distortion across subcarriers rather than concentrating it on individual symbols, effectively mitigating nonlinearity-induced errors and enhancing the overall system reliability [10].

Likewise, Cha et al. examined non-coherent on–off keying (OOK)-modulated data for body channel communication (BCC) using a single-input multiple-output (SIMO) receiver over fast-varying channels [11]. They applied probabilistic, deviation-based, and hybrid methods to estimate BER performance, revealing precise and reliable detection under challenging channel conditions. Additionally, they emphasized the applicability of orthogonal frequency division multiplexing (OFDM) for VLC, showing its ability to increase data rates while maintaining low BER. Their findings highlight OFDM’s strong potential to enable safe and efficient VLC systems and provide a solid foundation for hybrid modulation strategies in optical wireless communication (OWC) [11].

Recent contributions have extended machine learning-based compensation techniques in VLC–OFDM systems. For instance, an adaptive predistortion method combined with frequency–symbol spreading demonstrates noteworthy BER improvement under LED nonlinearity and multipath channels [12]. Deep neural networks have also shown to be effective for channel estimation in indoor VLC systems, outperforming classical estimators for DCO–OFDM and ACO–OFDM schemes [13]. To address both temporal- and frequency-domain impairments, hybrid equalization schemes have been proposed, combining time-domain and frequency-domain processing to mitigate LED nonlinearity and channel distortion [14]. Furthermore, a deep hybrid neural network (DHNN) combining DNN and LSTM architectures is proposed to jointly model nonlinear LED distortions and inter-symbol interference in a DCO–OFDM-based VLC system. By exploiting the nonlinear modeling capability of DNNs and the temporal memory of LSTMs, the proposed approach achieves faster convergence and consistently outperforms conventional CNN–LSTM equalizers in terms of equalization accuracy [15]. These developments support the viability of AI-driven equalization and validate the hybrid CNN–transformer architecture proposed in this work.

Most existing AI-assisted VLC receivers focus on single-modulation formats and do not consider adaptive bit loading or hybrid amplitude–timing modulation within a unified deep learning-based equalization framework.

#### Limitations and Research Gaps

Despite significant advances in VLC systems, several fundamental challenges remain, limiting their practical adoption. One primary limitation is the inherent nonlinearity of LEDs, which introduces distortion that degrades signal quality and overall system performance. Additionally, complex and hybrid modulation schemes, while capable of supporting higher data rates, are often difficult to implement effectively under realistic channel conditions. Issues such as multipath propagation, ambient light interference, and mobility-induced fluctuations introduce signal degradation that is not fully addressed by the existing methods.

While AI and ML techniques have improved robustness, they still fall short in fully compensating for nonlinear effects, timing jitter, and channel noise simultaneously, particularly in high-speed or adaptive communication scenarios that are relevant to 5G and beyond [16,17]. Moreover, current solutions typically focus on either signal-level compensation or channel adaptation but rarely integrate both in a unified framework that can handle LED nonlinearity, dynamic channel fluctuations, and spectral efficiency optimization concurrently. These gaps point to the need for holistic frameworks that combine advanced signal processing with deep learning-based adaptive equalization and modulation strategies.

### 1.2. Our Contributions

In response to the limitations of the existing VLC receivers and hybrid modulation schemes, this work introduces a unified framework that integrates classical signal processing with deep learning-based equalization. The proposed approach mitigates LED nonlinearities, adapts to fluctuating optical channels, and dynamically allocates modulation order based on instantaneous link quality. By combining the local feature extraction capability of CNNs with the long-range sequence modeling strengths of transformers, the system achieves robust symbol reconstruction across a wide range of SNR conditions.

The main contributions of this work are as follows:

1. We propose a dual-modulation architecture combining quadrature amplitude modulation (QAM) and pulse-position modulation (PPM), supported by a CNN– transformer equalizer. This design jointly addresses nonlinear distortion, multipath effects, and timing uncertainties, improving overall reliability in challenging indoor optical environments.
2. We develop and compare a CNN-based predistorter with traditional polynomial predistortion models. The learned predistorter achieves notably better nonlinear compensation, reducing QAM BER by 20–25% under moderate and strong LED nonlinearity, outperforming classical PD in both BER and mean squared error (MSE).
3. The framework integrates adaptive bit loading (ABL) to dynamically allocate modulation order per OFDM subcarrier according to instantaneous SNR. This adaptation increases spectral efficiency, yielding throughput improvements of approximately 15–16% compared to uniform 16-QAM loading.
4. We conduct an extensive analysis of QAM and PPM signaling, including pilot-spacing strategies, equalization performance, nonlinear distortion scenarios, and throughput trends, demonstrating robust model behavior across low-, moderate-, and high-SNR regimes.
5. We demonstrate that the proposed framework is particularly suitable for body area networks (BANs), where body shadowing, mobility-induced fluctuations, and strict power constraints demand highly adaptive nonlinearity-resilient VLC solutions. The aspects of hybrid modulation, learned equalization, and dynamic bit allocation synergistically address these constraints.

This work bridges classical VLC signal processing with modern AI-based compensation strategies, providing a scalable, adaptive, and robust solution for end-to-end optical communication in both indoor and body-centric scenarios. By explicitly addressing the key research gaps (LED nonlinearity, channel fluctuations, and modulation adaptability), our framework advances VLC system design toward more practical high-performance deployments.

The use of a hybrid 16-QAM and 4-PPM modulation scheme is motivated by the complementary strengths of the two formats. QAM, implemented over OFDM, provides high spectral efficiency and supports high-rate data transmission, but it is highly sensitive to LED nonlinearities and amplitude distortions. In contrast, PPM encodes information in the pulse position rather than amplitude, making it robust to clipping and power fluctuations, albeit with lower data-rate capability. By superimposing the two waveforms, the system simultaneously benefits from the high-throughput characteristics of QAM and the robustness of PPM, enabling reliable operation over a wide range of VLC channel conditions. This is particularly advantageous in BAN scenarios, where mobility, shadowing, and device orientation cause rapid fluctuations in received power. The hybrid formulation thus provides graceful performance degradation and improved resilience compared to single-modulation VLC schemes.

### 1.3. Paper Organization

The remainder of this paper is organized as follows. Section 2 presents the overall system model, including the transmitter architecture, LED nonlinearity, VLC channel, predistortion strategy, and CNN–transformer equalizer. Section 3 reports the simulation results for the proposed framework, examining the impact of predistortion, pilot spacing, deep learning equalization, and adaptive bit loading. Section 4 discusses the implications of these results and highlights the advantages and limitations of the proposed approach. Section 5 concludes the paper and outlines directions for future work.

## 2. Materials and Methods

This study investigates the performance of a VLC system employing a hybrid modulation strategy that combines 16-QAM and 4-PPM signals. The main objective is to assess how predistortion (PD) and LED nonlinearities, parameterized by the coefficient α, influence link reliability under realistic optical channel impairments. To ensure a comprehensive evaluation, multiple scenarios were generated by varying pilot spacing, nonlinear distortion levels, and channel configurations, allowing the system to be tested across representative VLC operating conditions. Performance metrics including BER, symbol error rate (SER), and MSE were computed for both modulation branches.

A further objective is to evaluate a hybrid CNN–TFN deep learning model designed to enhance signal recovery after transmission and equalization. The CNN–TFN architecture is adopted because it captures complementary feature domains: CNN layers extract local temporal structures and distortion signatures, while transformer layers model global dependencies across the signal sequence. This combination enables more effective equalization of nonlinear and frequency-selective optical channels compared to conventional or single-model approaches.

### 2.1. Problem Formulation

This work considers a hybrid VLC transmitter in which two parallel modulation branches generate a 16-QAM waveform $x_{\mathrm{QAM}}\left[n\right]$ and a 4-PPM waveform $x_{\mathrm{PPM}}\left[n\right]$. These discrete-time signals are combined at the sample level to form the composite transmit waveform

(1) $$s\left[n\right]=x_{\mathrm{QAM}}\left[n\right]+x_{\mathrm{PPM}}\left[n\right],$$

where *n* denotes the discrete-time sample index.

#### 2.1.1. LED Nonlinear Transmission Model

The LED introduces a nonlinear electro-optical transfer function that distorts the instantaneous intensity of the transmitted signal. The LED is modeled as a memoryless polynomial nonlinearity of order *K*:

(2) $$y\left[n\right]=f_{\alpha }\;\left(x[n]\right)=\sum_{k=1}^{K}a_{k}\left(\alpha \right)\;x^{k}\left[n\right],$$

where x[n] is the LED input sample, y[n] is the LED output sample, $a_{k}\left(\alpha \right)$ are the distortion coefficients, and α controls the severity of the nonlinearity. Larger values of α correspond to stronger amplitude compression and curvature in the LED transfer characteristic. The considered nonlinearity coefficients (α = 0.5, 1.0, and 1.8) are selected to represent mild, moderate, and strong LED distortion regimes, respectively, allowing systematic evaluation of predistortion effectiveness under progressively harsher operating conditions.

To mitigate this impairment, a predistorter (PD) $g_{P}\left(\cdot \right)$ approximating the inverse mapping of (2) is applied to the composite waveform s[n] prior to LED modulation:

(3) $$u\left[n\right]=g_{P}\;\left(s[n]\right)=\sum_{p=1}^{P}b_{p}\;s^{p}\left[n\right],$$

where u[n] is the predistorted signal and $\left\{b_{p}\right\}$ are the PD coefficients. The predistorted samples are then scaled and biased to match the LED’s admissible operating range:

(4) x[n]=Gu[n]+B,

with *G* and *B* denoting the gain and DC bias, respectively.

#### 2.1.2. Optical Channel Model

After LED modulation, the waveform propagates through an optical channel characterized by a flat memoryless gain. The total optical gain is computed as the sum of the line-of-sight (LOS) and first-order non-line-of-sight (NLOS) reflection components:

(5) $$H_{\mathrm{Total}}=H_{\mathrm{LOS}}+H_{\mathrm{NLOS}}.$$

The received electrical signal is modeled as

(6) $$r\left[n\right]=H_{\mathrm{Total}}\;y\left[n\right]+w\left[n\right],$$

where $w\left[n\right]∼N\left(0,\sigma_{w}^{2}\right)$ is additive Gaussian noise representing the combined effects of thermal noise, shot noise, and ambient light fluctuations. This memoryless channel model reflects the short-range nature of BAN-oriented VLC links. The adoption of a memoryless channel model is motivated by the short-range nature of body area network (BAN) VLC links, where the transmitter–receiver distance is limited and the line-of-sight component dominates the received signal. Under these conditions, the channel delay spread is typically much smaller than the symbol duration, and inter-symbol interference due to multipath propagation can be neglected. As a result, a memoryless gain model provides an accurate first-order representation of the optical channel for the considered scenario.

#### 2.1.3. Receiver Model and Learning Objective

The receiver aims to recover the transmitted symbol sequence from the noisy nonlinearly distorted observations r[n]. Let $s_{m}$ denote the *m*-th transmitted symbol (QAM or PPM), and let ${\hat{s}}_{m}$ denote its estimate. The equalization operation is modeled as a function

(7) $${\hat{s}}_{m}=D\left(r[n]\right),$$

where D(·) is implemented by the proposed hybrid CNN–TFN architecture.

The equalizer is trained to minimize the mean squared reconstruction error:

(8) $$\min_{\Theta }\;E\;\left[\;∥s_{m}-{\hat{s}}_{m}{∥}^{2}\right],$$

where Θ collects all learnable neural network parameters.

#### 2.1.4. Adaptive Bit Loading

For the OFDM-based QAM branch, an adaptive bit loading (ABL) rule is used to select the number of bits assigned to each subcarrier. Let *i* denote the subcarrier index and ${\mathrm{SNR}}_{i}$ its instantaneous SNR. A simple SNR-threshold scheme is adopted:

(9) $$m_{i}=\left\{\begin{array}{cc} 6, & {\mathrm{SNR}}_{i}\geq \gamma_{64\mathrm{QAM}},\\ 4, & {\mathrm{SNR}}_{i}<\gamma_{64\mathrm{QAM}}, \end{array}\right.$$

where $m_{i}$ is the number of bits carried by subcarrier *i*, and $\gamma_{64\mathrm{QAM}}$ is the SNR threshold required for reliable 64-QAM transmission (set in the simulations according to the target BER).

### 2.2. System Architecture

**Figure 1:**
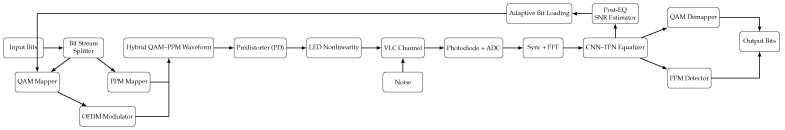
System-level block diagram of the proposed hybrid QAM–PPM VLC–OFDM framework. Hybrid modulation and predistortion are applied at the transmitter, CNN–TFN equalization compensates channel and hardware impairments at the receiver, and adaptive bit loading adjusts the QAM modulation order based on post-equalization SNR estimates.

Figure 1 summarizes the end-to-end processing chain of the proposed framework and clarifies the roles of hybrid modulation, predistortion, CNN–TFN equalization, and the ABL feedback loop within a single transmitter–receiver architecture.

**Figure 2:**
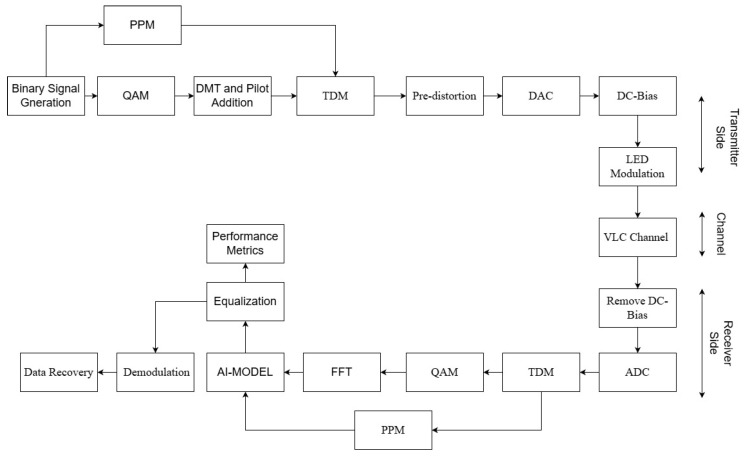
System architecture and hybrid modulation processing.

A complete VLC simulation environment was developed to evaluate the proposed hybrid modulation and equalization framework. The system architecture is organized into four main processing stages: (i) signal generation, (ii) nonlinear optical transmission, (iii) channel propagation, and (iv) receiver-side demodulation and equalization. A block diagram of the system is shown in Figure 2.

The ordering of the processing blocks in the proposed architecture is guided by physical-layer causality and practical implementation considerations. Predistortion is applied at the transmitter to compensate LED nonlinearity prior to optical modulation, where such impairments originate and can be most effectively mitigated. After propagation through the VLC channel, the CNN–transformer equalizer operates at the receiver to compensate residual nonlinear distortion, channel attenuation, noise, and timing effects that cannot be fully addressed by transmitter-side processing. Adaptive bit loading is performed after equalization as it relies on post-equalization SNR estimates to reliably assign modulation orders to individual subcarriers. This sequential design ensures that each processing stage operates on the most informative signal representation available.

Independent binary sequences are mapped onto 16-QAM and 4-PPM symbols. For the QAM branch, discrete multi-tone (DMT) processing with Hermitian symmetry is applied to generate a real-valued OFDM waveform that is compatible with intensity modulation. The PPM branch generates real-valued non-negative pulses that naturally meet IM/DD constraints. The two waveforms are sample-wise added to form the hybrid baseband signal s[n].

The hybrid waveform s[n] is processed by the polynomial predistorter (PD) $g_{P}\left(\cdot \right)$ (Section 2.3) to mitigate LED nonlinearities. After predistortion, the signal is scaled and biased according to (4), ensuring operation within the LED’s linear region. The biased waveform x[n] is then passed through the nonlinear LED transfer function $f_{\alpha }\left(\cdot \right)$ to produce the optical output y[n], as described in (2).

The optical waveform y[n] propagates through a short-range VLC channel characterized by a total gain $H_{\mathrm{Total}}$ and additive noise, yielding the received samples as in (6). The derivation of $H_{\mathrm{Total}}$ from LOS and NLOS components, as well as the noise statistics, is detailed in Section 2.4.

At the receiver, the electrical signal is digitized, DC bias is removed, and the hybrid signal is separated into QAM and PPM branches using timing-based demultiplexing. The QAM branch undergoes FFT demodulation, pilot-assisted equalization, and optional adaptive bit loading (Section 2.1). The PPM branch is processed directly in the time domain. Both separated branches are fed to the CNN–TFN equalizer (Section 2.5), which estimates the transmitted symbol sequences ${\hat{s}}_{m}$ for QAM and PPM, respectively. Error rate metrics (BER, SER, and MSE) are computed by comparing ${\hat{s}}_{m}$ with the ground-truth symbols.

### 2.3. Predistorter (PD)

Commercial LEDs exhibit a nonlinear electro-optical transfer characteristic that distorts the instantaneous intensity of the transmitted waveform, degrading the performance of high-order and hybrid modulation formats. To compensate for this impairment, a PD is applied at the transmitter to approximate the inverse of the LED nonlinearity.

The LED is modeled as a memoryless polynomial nonlinearity of order *K* acting on the LED input samples x[n], as shown in (2), where y[n] is the LED output, the coefficients $a_{k}\left(\alpha \right)$ describe the device’s nonlinear characteristic, and the parameter α controls the distortion severity. Let s[n] denote the composite hybrid QAM–PPM signal prior to predistortion. The PD is implemented as a polynomial of order *P*, as shown in (3), where u[n] is the predistorted signal and $\left\{b_{p}\right\}$ are the PD coefficients. Before entering the LED, the predistorted samples are scaled and biased to ensure operation within the valid LED range, as in (4), where x[n] denotes the LED input signal, *G* is the modulation gain, and *B* is the DC bias that prevents LED cutoff and saturation.

The cascade of PD and LED results in the nonlinear mapping

(10) $$z\left[n\right]=f_{\alpha }\left(x\left[n\right]\right)=f_{\alpha }\;\left(G\;u[n]+B\right),$$

where z[n] is the optical-domain signal injected into the VLC channel.

The PD coefficients are chosen to make the overall PD–LED cascade approximately linear

(11) $$z\left[n\right]\approx G_{\mathrm{eff}}\cdot s\left[n\right],$$

for a desired effective gain $G_{\mathrm{eff}}$. This leads to the nonlinear least-squares optimization:

(12) $$b^{★}=\arg\min_{b}E\;\left[{\left|f_{\alpha }\;\left(G\;g_{P}\left(s\left[n\right];b\right)+B\right)-G_{\mathrm{eff}}\;s\left[n\right]\right|}^{2}\right],$$

where $b={\left[b_{1},\ldots ,b_{P}\right]}^{T}$ and ^T^ is the transpose operator.

By varying *P* and α, the influence of PD complexity and LED nonlinearity on distortion mitigation can be systematically evaluated. Lower-order PDs mainly compensate global gain and curvature, whereas higher-order PDs more accurately approximate the inverse LED characteristic, improving linearity for both QAM and PPM branches.

### 2.4. VLC Channel Modeling and System Parameters

The VLC channel is modeled using a short-range configuration representative of a typical indoor BAN scenario, where a wearable photodetector receives optical signals from a ceiling-mounted LED luminaire. This setup captures the dominant link characteristics encountered in practical BAN environments, including line-of-sight (LOS) signal dominance, reflections from nearby surfaces, and ambient-light-induced noise.

Under the adopted VLC channel model, the received discrete-time electrical signal can be expressed as

(13) $$r\left[n\right]=H_{\mathrm{Total}}y\left[n\right]+w\left[n\right],$$

where $H_{\mathrm{Total}}$ denotes the overall optical channel gain, accounting for both line-of-sight (LOS) and first-order non-line-of-sight (NLOS) components, and w[n] represents additive noise. In the considered body area network (BAN) scenario, the transmitter–receiver distance is short and the LOS path dominates the received signal, allowing the optical channel to be accurately modeled as a memoryless gain. The total channel gain is expressed as $H_{\mathrm{Total}}=H_{\mathrm{LOS}}+H_{\mathrm{NLOS}}$, while the noise term is modeled as a zero-mean Gaussian random process with variance $\sigma_{w}^{2}$. Detailed expressions for the LOS and NLOS channel gains, as well as for the noise variance computation, follow standard VLC formulations and are provided in Appendix A for completeness.

The total channel gain used for simulation and equalization is the sum of LOS and NLOS contributions, as in (5).

The numerical values obtained for $H_{\mathrm{LOS}}$, $H_{\mathrm{NLOS}}$, and $H_{\mathrm{Total}}$ are reported in Table 1.

As expected in short-range indoor BAN scenarios, the LOS component dominates, while the NLOS reflection introduces a smaller but non-negligible contribution to the overall link budget.

The simulation parameters reported in Table 2 are selected to represent a realistic indoor VLC scenario for body area networks. The transmitter–receiver distance of 2 m and Lambertian order ℓ=1 correspond to a ceiling-mounted LED luminaire illuminating a wearable photodetector, as commonly assumed in indoor BAN-oriented VLC studies. The photodetector area, responsivity, noise bandwidth, and ambient irradiance values are chosen to reflect typical commercial VLC front-end characteristics reported in the literature. These parameter choices ensure that the simulated channel conditions are representative of practical short-range VLC deployments while allowing observable nonlinear and noise-induced impairments.

### 2.5. CNN–TFN Model

The CNN–transformer network operates purely as a signal equalization and symbol estimation block within the VLC receiver. Its output is used exclusively for demodulation and performance evaluation, and it does not perform advisory or decision-making functions related to security, authentication, or access control.

To compensate for the combined effects of LED nonlinearities, channel attenuation, and additive noise, a hybrid convolutional neural network–transformer (CNN–TFN) architecture is adopted at the receiver. The goal of the model is to estimate the transmitted symbol sequence from the distorted and noisy received samples r[n] by learning an effective inverse mapping

$$r\left[n\right]\;⟼\;{\hat{s}}_{m},$$

as introduced in Section 2.1.

The received time-domain signal r[n] is segmented into frames of fixed length, which serve as input sequences to the neural equalizer. For the QAM branch, the real and imaginary parts of the time-domain OFDM signal are treated as separate input channels, thereby preserving the complex-valued structure of the modulation. For the PPM branch, the input consists of a single real-valued sequence capturing the pulse-position information.

Prior to being fed to the network, each frame is normalized to zero mean and unit variance to stabilize training and to reduce the influence of absolute power variations caused by changes in SNR or channel gain.

The first stage of the architecture is a one-dimensional CNN front-end designed to extract local temporal features from the received sequence. This block consists of a stack of 1D convolutional layers with small kernel sizes, each followed by ReLU activation, batch normalization, and dropout. The convolutional filters capture short-range distortion patterns associated with LED nonlinearities, residual inter-symbol interference, and local perturbations introduced by the PD–LED cascade.

Let $r\in R^{L\times C_{\mathrm{in}}}$ denote an input frame of length *L* with $C_{\mathrm{in}}$ channels. The CNN front-end produces a feature sequence $F_{\mathrm{CNN}}\in R^{L\times C_{\mathrm{feat}}}$ that preserves the temporal dimension while increasing the feature dimensionality through multiple convolutional layers.

The CNN features are then processed by a transformer network (TFN) block that models long-range dependencies across the frame. The TFN employs Multi-Head Self-Attention (MHSA) layers to compute context-aware representations, allowing each time step to attend to all others within the frame. This is particularly beneficial for OFDM-based QAM signals, where frequency-selective attenuation and nonlinear distortion introduce dependencies that extend beyond the local neighborhood captured by CNN filters.

Positional encodings are added to the CNN feature sequence to retain information about relative sample ordering. The TFN block is composed of a stack of MHSA layers, each followed by layer normalization and a position-wise feed-forward network with ReLU activation. Residual connections are used throughout the transformer block to facilitate gradient flow and improve training stability.

The output of the transformer block is passed through one or more fully connected layers to produce the final symbol estimates. For the QAM equalizer, the network outputs the real and imaginary components of the estimated symbols ${\hat{s}}_{m}$, which are then mapped to the nearest constellation points. For the PPM equalizer, the network outputs a probability distribution over the possible pulse positions, and the most likely position is selected as the symbol estimate.

Both QAM and PPM models are trained using a mean squared error loss between the true symbols $s_{m}$ and the predicted symbols ${\hat{s}}_{m}$, as depicted in (8), where Θ denotes the set of all trainable parameters of the CNN–TFN architecture. The Adam optimizer is used for training with mini-batches of input frames, and early stopping is applied based on validation performance to prevent overfitting.

It is important to note that the proposed CNN–transformer equalizer is trained to extract features related to physical-layer signal impairments, including LED-induced nonlinearity, channel attenuation, noise, and timing distortion. No explicit threat or adversarial model is considered. Accordingly, the learned representations should be interpreted as impairment-aware features rather than threat-aware or security-oriented features.

#### Hybrid Architecture Motivation

The choice of a hybrid CNN–TFN architecture is motivated by the complementary properties of the two components. The CNN front-end efficiently captures local temporal features related to LED-induced nonlinear distortions and short-range channel effects, while the transformer block exploits long-range temporal and spectral correlations that arise in OFDM-based hybrid VLC systems. As shown in Section 3, this combination yields improved symbol reconstruction accuracy for both QAM and PPM branches compared to using either convolutional or attention-based models alone.

In the QAM equalizer, a hybrid CNN–TFN architecture is adopted to jointly capture local temporal distortions and long-range symbol dependencies, as summarized in Table 3. The CNN front-end consists of three Conv1D layers, each operating with a stride of 1 and same padding to preserve symbol length and temporal alignment. This design choice avoids temporal downsampling, which is critical for QAM symbol recovery, where both amplitude and phase information are continuously distributed over time.

The first Conv1D layer expands the input waveform into 64 feature channels, providing sufficient capacity to capture low-level nonlinear distortions introduced by LED transfer characteristics. This is followed by a deeper convolution that increases the channel dimension to 128, enabling the model to learn higher-order temporal patterns and inter-sample correlations associated with dispersive VLC channels. A third lightweight convolution with the same channel dimension is employed to refine the extracted features while keeping the overall parameter count moderate. ReLU activation follows each convolution to introduce nonlinearity, and max pooling is intentionally omitted to retain full temporal resolution at the symbol level.

The resulting CNN features are then passed to the transformer network encoder with a model dimension of 128, matching the CNN output to avoid additional projection overhead. A sinusoidal positional encoding is added to explicitly encode symbol timing information prior to attention processing, which is essential for resolving inter-symbol interference. The transformer consists of two encoder blocks, selected as a balanced trade-off between modeling long-range dependencies and maintaining low inference latency for real-time VLC applications.

Each encoder block employs an MHSA to capture global temporal interactions, followed by an FFN that expands the feature dimension from 128 to 256 and compresses it back to 128. This expansion–compression strategy increases representational capacity while controlling computational complexity. Pre-LayerNorm and dropout are incorporated to stabilize training and improve generalization under varying channel conditions.

This combined CNN–TFN structure enables effective modeling of both short-range nonlinear distortions and long-range inter-symbol interference inherent in QAM-based VLC systems. After the second encoder block, a linear projection maps the 128-dimensional transformer output to a two-dimensional I/Q representation, yielding a regression-style output with one complex symbol per time step. The final symbol decisions are obtained by mapping the predicted I/Q pairs to the nearest QAM constellation point. Overall, the architecture preserves temporal fidelity, effectively mitigates nonlinear and dispersive channel effects, and remains computationally efficient for practical real-time VLC implementations.

For the PPM signal model, a reduced-complexity CNN–TFN architecture is employed, as shown in Table 4. Unlike QAM, PPM symbols are characterized by sparse high-energy pulses, where the discriminative information lies primarily in the temporal position of the pulse rather than the detailed waveform shape. Accordingly, the architecture is optimized for accurate pulse-position detection and symbol index classification.

The front-end consists of a single Conv1D layer that expands the received waveform into 64 feature channels with an output shape of (batch, 64, 128). A stride of 1 and same padding are used to preserve the temporal structure of the signal, which is crucial for precise localization of the pulse within the symbol duration. A ReLU activation follows to introduce nonlinearity, and sinusoidal positional encoding is added to explicitly inject temporal ordering information required for effective transformer-based processing of sparse PPM features.

The encoded sequence is then processed by an MHSA layer with four attention heads, enabling the model to capture temporal shifts and spreading effects caused by LED nonlinearity and multipath propagation. The subsequent feed-forward stage expands the feature dimension from 64 to 128 to extract richer hidden representations, followed by dropout and LayerNorm to improve regularization and training stability. A transformer encoder block further refines the temporal representations through an additional cycle of attention and FFN operations, which is sufficient given the simpler temporal structure of PPM signals.

To convert the sequence-level representation into a symbol-level prediction, a Global Average Pooling (GAP) operation is applied across the temporal dimension, producing a compact (batch, 128) embedding that summarizes pulse-related evidence across the entire frame. This pooling strategy is well-suited for PPM as symbol decisions depend on global pulse position rather than per-sample regression. Finally, a linear output layer projects the pooled representation into the PPM symbol space, yielding a probability distribution over all possible pulse positions. The detected symbol corresponds to the argmax of this distribution.

From a computational perspective, the depth and width of the proposed CNN– transformer architecture are intentionally limited to balance equalization performance and inference complexity. The QAM branch employs a modest number of convolutional and attention layers to capture nonlinear and long-range dependencies, while the PPM branch adopts a reduced-depth architecture reflecting the simpler temporal structure of PPM signals. This design choice limits the number of trainable parameters and dominant operations, making the proposed equalizer compatible with VLC receivers equipped with moderate processing capability, as typically envisioned in body area network scenarios.

## 3. Results

This section presents a concise description of the experimental results, their interpretation, and the conclusions that can be drawn.

### 3.1. Nonlinearity Effect and Predistorter (PD) Analysis

The hybrid signal is initially normalized to the range [−1, 1]. Nonlinearity is modeled using a cubic function with coefficients α=0.5,1.0, and 1.8. The study focuses on how different levels of nonlinearity affect the hybrid signal and the effectiveness of PD strategies. The signal is passed through the PD, which estimates the inverse coefficients corresponding to α, thereby compensating for nonlinear distortions and preserving the signal structure.

#### 3.1.1. Analysis of Third-Order PD with α=1.0

**Figure 3:**
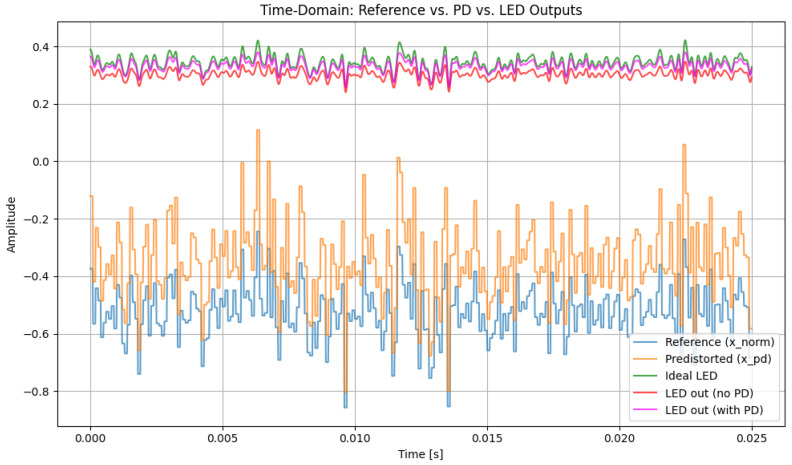
Time-domain waveform analysis of the hybrid signal with 3rd-order PD and α=1.0, illustrating the signal behavior under nonlinear distortion mitigation.

Figure 3 compares the time-domain hybrid 16-QAM/4-PPM waveform at the LED output for the ideal (reference) case, for the nonlinear LED without predistortion, and for the nonlinear LED with a third-order PD (α = 1.0). Without PD, the LED introduces clear amplitude compression, especially around the waveform peaks, causing the hybrid signal to deviate from the reference. When the third-order PD is applied, the resulting waveform closely follows the ideal one, with peaks and valleys partially restored while the PPM pulse positions are preserved, indicating effective mitigation of moderate LED nonlinearity.

**Figure 4:**
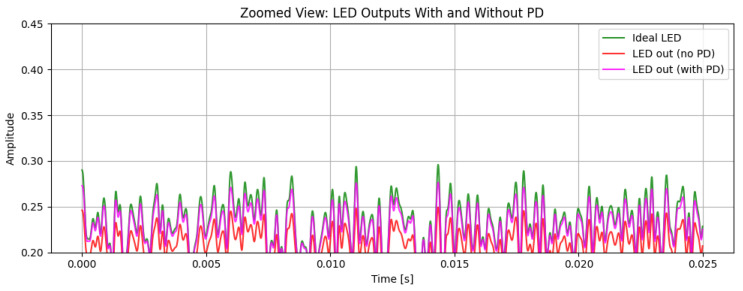
Magnified image of the LED hybrid signal with 3rd-order PD and α=1.0, illustrating the signal behavior under nonlinear distortion mitigation.

Figure 4 shows the magnified section for better observation of the PD effect in the LED signal in our system. The observation shows us the ideal LED signal (green) where no nonlinearity is applied and is an ideal signal, the LED signal with a nonlinearity effect along with PD (pink), and the LED signal with a nonlinearity effect without PD (red). The signal with no PD shows greater signs of distortion effects, with amplitude compression across the waveform. The distortion can be seen significantly in the local maxima and local minima, where the LED’s current–luminance relationship passes to the nonlinear working region. This is proved as the ideal LED signal, and LED signals without PD seem far apart compared to the ideal LED signal and LED signal with PD. Conversely, for LED signals with PD, the current–luminance characteristics are compensated and hence maintain the properties of the ideal LED signal, showing improved peak restoration and reduced waveform deformation. This shows that the PD successfully counteracts the LED’s nonlinear transfer behavior, maintaining the intended amplitude structure and enhancing the overall signal integrity in the optical domain.

After predistortion, the hybrid QAM–PPM waveform retains a structure that is much closer to the ideal reference, confirming that the cubic PD effectively mitigates the dominant nonlinear distortion components. A DC bias of 0.5 and a gain of 0.3 are applied to ensure that the signal operates within the permissible range of the LED, avoiding clipping or saturation.

**Figure 5:**
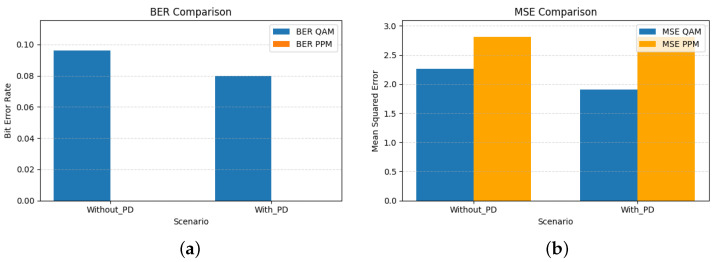
(**a**) BER comparison and (**b**) MSE comparison for QAM and PPM signals with 3rd-order PD and α=1.0. The results demonstrate the effectiveness of PD for nonlinear distortion mitigation in QAM signals, while PPM remains largely unaffected.

The bar graphs shown in Figure 5 illustrate the complementary behavior of QAM and PPM signals in the hybrid VLC system. QAM, being sensitive to amplitude and phase nonlinearities, benefits significantly from the third-order PD, achieving lower BER and MSE values. In contrast, PPM remains largely unaffected by nonlinear distortion, highlighting its inherent robustness against LED nonlinearity. The numerical results corresponding to these trends are summarized in Table 5. The BER of QAM decreases from 0.0962 to 0.0800, and MSE reduces from 2.269 to 1.909, indicating that QAM is sensitive to amplitude nonlinearities, where amplitude compression directly affects constellation geometry and increases bit error. By linearizing the transfer characteristics of the LED, the PD restores the intended amplitude spacing of the QAM symbols, resulting in measurable gains in both BER and MSE.

#### 3.1.2. Analysis of Fifth-Order PD with α=0.5

To further investigate the hybrid signal behavior, the PD order and nonlinearity coefficient α were adjusted while maintaining the same DC bias and gain to keep the signal within the LED’s operating range. Increasing the PD order allows the PD to better follow the nonlinear characteristics of the LED, resulting in a waveform that is closer to the ideal reference signal.

**Figure 6:**
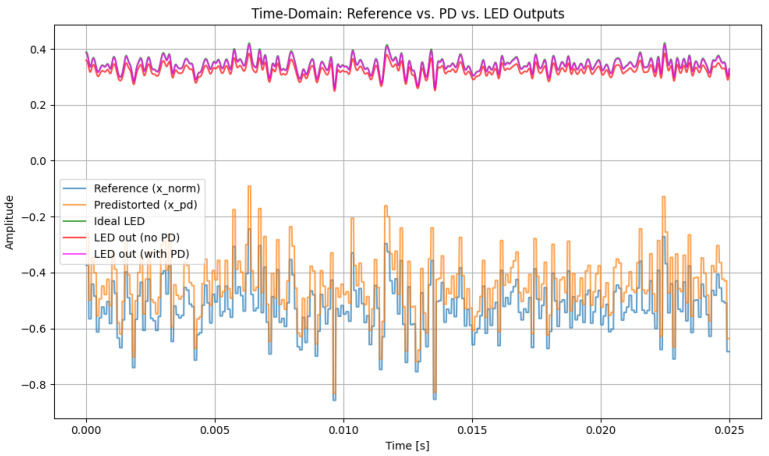
Time-domain waveform analysis of the hybrid signal with 5th-order PD and α=0.5. The increased PD order closely tracks the reference signal, improving QAM signal linearity.

Figure 6 shows the time-domain hybrid waveform after applying a fifth-order PD under mild LED nonlinearity (α=0.5). In this regime, the LED distortion is limited, and the fifth-order PD is able to closely replicate the ideal LED response. The PD-compensated waveform aligns more tightly with the reference signal than the uncompensated waveform, with reduced amplitude deformation and more accurate reproduction of local maxima and minima. This behavior confirms that increasing the PD order enhances the linearization accuracy when the LED operates under weak nonlinear conditions, leading to a transmitted optical waveform that closely matches the intended hybrid 16-QAM/4-PPM structure.

**Figure 7:**
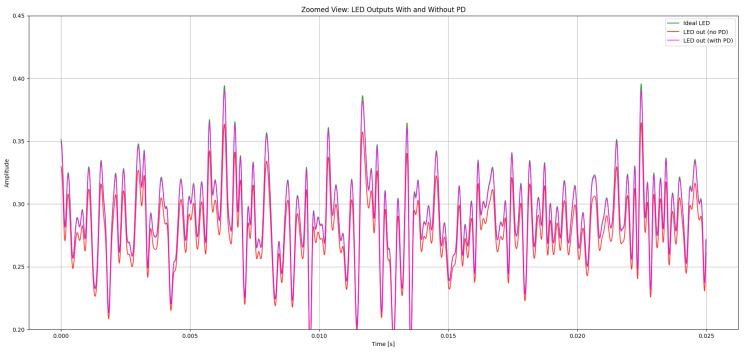
Magnified image of the LED hybrid signal with 5th-order PD and α=0.5, illustrating the signal behavior under nonlinear distortion mitigation.

Figure 7 provides a magnified view of the LED output under different PD orders and nonlinearity levels. As the PD order increases, the compensated LED waveform becomes progressively closer to the ideal reference, exhibiting reduced amplitude deformation and improved peak fidelity. This trend is consistent with the behavior observed for the third-order case.

**Figure 8:**
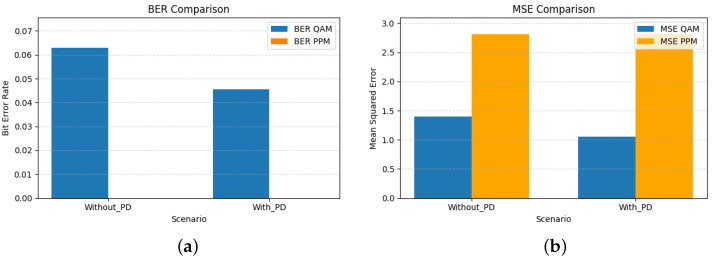
(**a**) BER comparison and (**b**) MSE comparison for QAM and PPM signals with 5th-order PD and α=0.5. Higher PD order improves QAM signal performance, while PPM remains unaffected.

Figure 8 complements this analysis by quantifying the resulting BER and MSE for both modulation branches. As expected, higher-order PD significantly improves the QAM performance, while the PPM branch remains essentially unaffected due to its inherent robustness to amplitude distortion.

Table 6 summarizes these improvements: for α=0.5, the QAM BER decreases from 0.0629 to 0.0456 and the MSE from 1.395 to 1.051 when the fifth-order PD is applied. These results confirm that increasing the PD order enhances the linearization of the LED response in mild nonlinearity regimes, yielding a waveform that more closely matches the intended hybrid structure without altering PPM performance. For the DC-based equalization approach applied to the PPM signal, a total of 83 four-PPM symbols were evaluated, corresponding to 166 transmitted bits for an input size of (83, 128). As a result, the measurable BER is <6.02 $\times 10^{-3}$, i.e., 0 errors over 166 bits. It is important to distinguish between the measured BER and the minimum BER that can be reliably estimated from a finite dataset. When the equalizer produces zero bit errors over $N_{\mathrm{bits}}$ transmitted bits, the reported BER is numerically zero, but the true BER of the system can only be stated to be smaller than $1/N_{\mathrm{bits}}$. In our experiments, the PPM evaluation set contains 166 bits, which implies a minimum measurable BER of $6.02\times 10^{-3}$. Therefore, cases reported with BER=0 should be interpreted as “no errors observed” rather than implying an error-free channel.

#### 3.1.3. Analysis of Seventh-Order PD with α=1.8

In this scenario, the LED nonlinearity coefficient is increased to α=1.8 to emulate severe distortion conditions. Such strong nonlinearity affects not only the QAM amplitude structure but also the PPM pulse timing. A DC bias of 0.4 and a gain of 0.3 are applied to ensure that the predistorted waveform remains within the valid LED operating region, preventing saturation and cutoff while preserving sufficient modulation depth.

**Figure 9:**
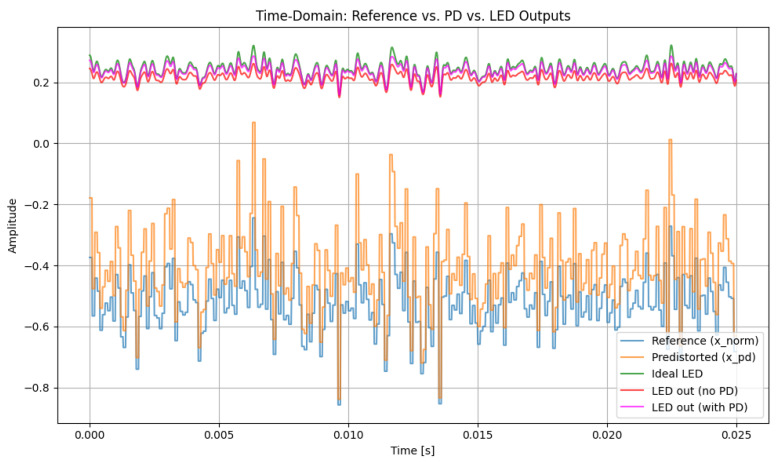
Time-domain waveform analysis of the hybrid signal with 7th-order PD and α=1.8, showing how the higher-order PD mitigates nonlinear distortions for both QAM and PPM signals.

Figure 9 shows the time-domain hybrid waveform at the LED output with and without the seventh-order PD. The uncompensated waveform exhibits significant amplitude compression and deformation due to the high nonlinearity level. In contrast, the PD-compensated signal aligns more closely with the ideal reference, demonstrating effective suppression of nonlinear distortion even in this high-α regime.

**Figure 10:**
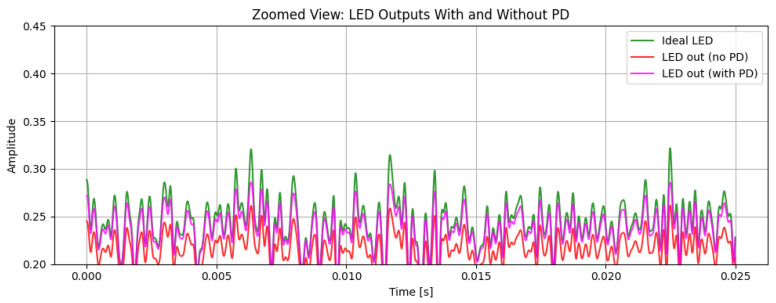
Magnified image of the LED hybrid signal with 7th-order PD and α=1.8, illustrating the signal behavior under nonlinear distortion mitigation.

A magnified view in Figure 10 highlights the improvement more clearly. The PD-corrected waveform exhibits restored peak amplitudes and smoother transitions, while the uncompensated waveform shows visibly sharper distortions and degraded pulse structure. These observations confirm that increasing the PD order enhances the linearization accuracy under strong LED nonlinearities.

**Figure 11:**
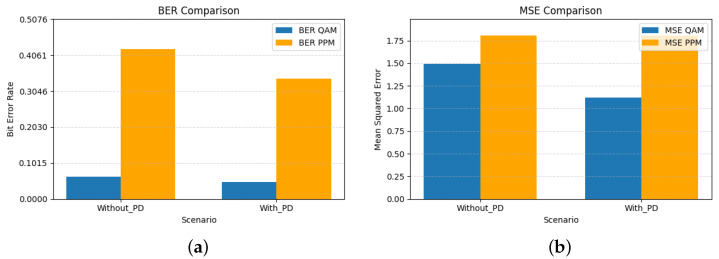
(**a**) 16-QAM BER comparison and (**b**) MSE comparison for QAM and PPM signals with 7th-order PD and α=1.8. The results demonstrate improvements in both QAM and PPM signal performance due to higher-order PD.

The performance impact of the seventh-order PD is quantified in Figure 11. As expected, both BER and MSE decrease for the QAM branch, confirming that the higher-order PD more effectively compensates the amplitude and phase distortions introduced by the LED. The PPM branch also shows a reduction in raw BER, from 0.4230 to 0.3406 (Table 7), indicating that the PD partially restores the shape and timing of the transmitted pulse. However, this BER level remains far too high for reliable detection and is presented here only as a baseline measurement under extreme nonlinearity (α=1.8). The MSE for PPM remains essentially unchanged, which is consistent with the fact that PPM errors are dominated by timing misalignment rather than amplitude distortion.

These results demonstrate that, under strong nonlinearities, high-order PD is essential for preserving the integrity of both modulation branches. QAM benefits from restored constellation amplitude spacing, while PPM gains robustness due to improved preservation of pulse peaks.

A detailed numerical comparison of BER and MSE values for different PD orders and LED nonlinearity levels is provided in Appendix B.

#### 3.1.4. Channel Impairments in VLC Systems

Table 1 reports the numerical values of the LOS, NLOS, and total optical channel gains used in the simulations. As expected in short-range indoor VLC links, the LOS component dominates the received power, while the first-order NLOS reflection contributes a smaller but non-negligible fraction of the total gain. Although the VLC channel is modeled as memoryless (Section 2.4), the presence of NLOS energy slightly increases the effective received power and therefore impacts the resulting SNR.

The table also summarizes the corresponding signal and noise power values and the resulting electrical SNR at the receiver. These values confirm that, under the selected BAN scenario and system parameters, the received SNR remains sufficiently high to enable meaningful evaluation of nonlinear distortion effects and predistortion compensation. Higher-order PDs therefore operate in a regime where both LED nonlinearity and channel noise influence BER and MSE performance, as analyzed in the following subsections.

#### 3.1.5. Comparison of a Simple PD and a CNN-Based PD

A comparative analysis was carried out between a conventional polynomial PD and a CNN-based PD. A fifth-order PD with a pilot spacing of 8, a DC bias of 0.4, and a gain of 0.3 was employed to ensure that the LED operated within its linear region while preserving sufficient modulation depth. The same channel and system parameters of Table 2 were used for both approaches to guarantee a fair comparison.

For the simple PD, the resulting BER and MSE values are reported in Table 8. As expected, QAM benefits from linearization, achieving a BER of 0.0483 and an MSE of 1.138. In contrast, the PPM branch remains largely unchanged, with BER = 0.3516 and MSE = 1.806, reflecting its inherent robustness to amplitude-based distortions due to its energy-detection nature.

The same evaluation was conducted using a CNN-based PD designed to learn a data-driven approximation of the inverse LED characteristic. The neural model was trained for 100 epochs using a learning rate of $1\times 10^{-3}$, a batch size of 64, and a lightweight architecture consisting of 2–3 convolutional layers. These settings were sufficient to capture the relevant local and temporal distortion patterns present in both QAM and PPM signals. The evolution of BER and MSE over training epochs is shown in Table 9.

The results indicate that the CNN-based PD provides a slight but measurable improvement over the conventional PD. For instance, the QAM BER decreases from 0.0483 to 0.0475 (approximately 1.6%), and the corresponding MSE decreases from 1.138 to 1.137. PPM also shows minor fluctuations across epochs, with its BER reaching a minimum of 0.3400 at epoch 90. These improvements arise because the neural PD can adaptively learn subtle nonlinear relationships that are difficult to approximate with a fixed polynomial model. Such data-driven compensation becomes increasingly valuable in scenarios where LED nonlinearities or operating conditions deviate from idealized polynomial assumptions.

### 3.2. Effect of PD on System Performance

**Figure 12:**
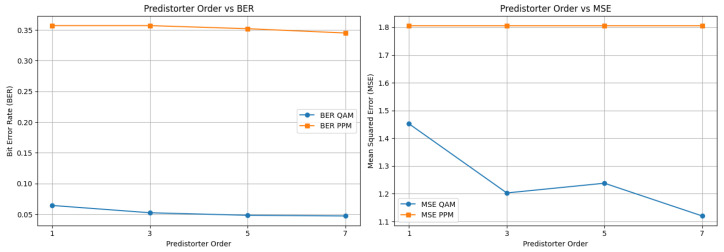
Impact of PD order on the BER performance of the hybrid VLC system under strong LED nonlinearity (α=1.8). Higher PD orders yield progressively lower BER for the QAM branch, with diminishing performance gains beyond the 5th order.

This simulation evaluates the performance of the VLC system as a function of the PD order and quantifies the impact of increasingly complex predistortion on system accuracy. Table 10 reports the BER and MSE obtained for different PD orders, while Figure 12 illustrates the corresponding BER trends.

As shown in the figure, increasing the PD order consistently improves QAM performance, with a noticeable reduction in BER from the first to the third and fifth orders. Beyond this point, however, the performance gain becomes marginal: the seventh-order PD offers only slight improvements over the fifth-order case. This diminishing return occurs because higher-order PDs more accurately approximate the LED nonlinear transfer function, but the additional polynomial terms contribute progressively smaller corrections to the overall distortion.

Considering both performance and computational cost, the fifth-order PD represents the most effective compromise. It provides nearly the same BER and MSE improvement as the seventh-order PD while requiring substantially fewer operations, making it the preferred configuration for hybrid QAM–PPM transmission under the tested nonlinearity conditions.

### 3.3. Pilot Spacing Analysis

The evaluated pilot spacings and SNR values are chosen to span the typical operating conditions of indoor VLC systems, enabling analysis of the trade-off between channel estimation accuracy and spectral efficiency under both moderate- and high-SNR regimes.

Pilot spacing directly influences the channel estimation accuracy, equalization quality, and overall spectral efficiency in the QAM branch of the hybrid VLC system. In this analysis, pilot intervals of 8, 16, 24, and 25 subcarriers were evaluated under identical channel conditions. All the simulations were performed with α=1.8, a DC bias of 0.4, and a gain of 0.3 to emulate strong LED nonlinearity and ensure consistent comparison across pilot configurations.

**Figure 13:**
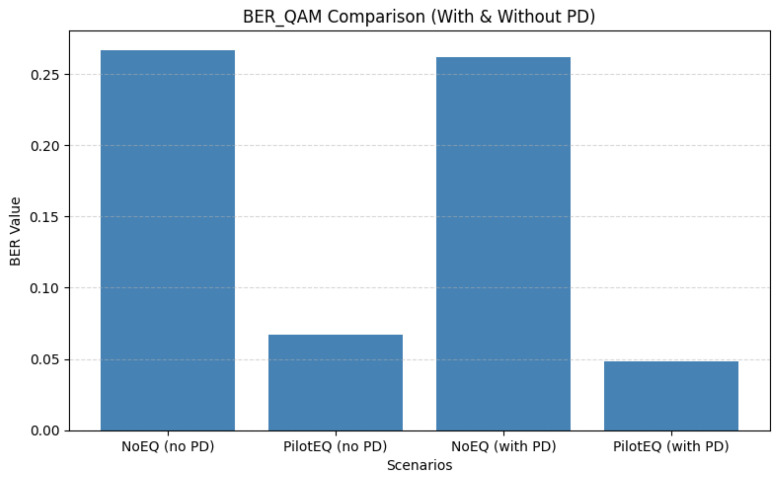
BER performance for the QAM branch with pilot spacing of 8. Dense pilot insertion improves channel estimation accuracy, resulting in significantly lower BER compared to the no-pilot scenario both with and without predistortion.

**Figure 14:**
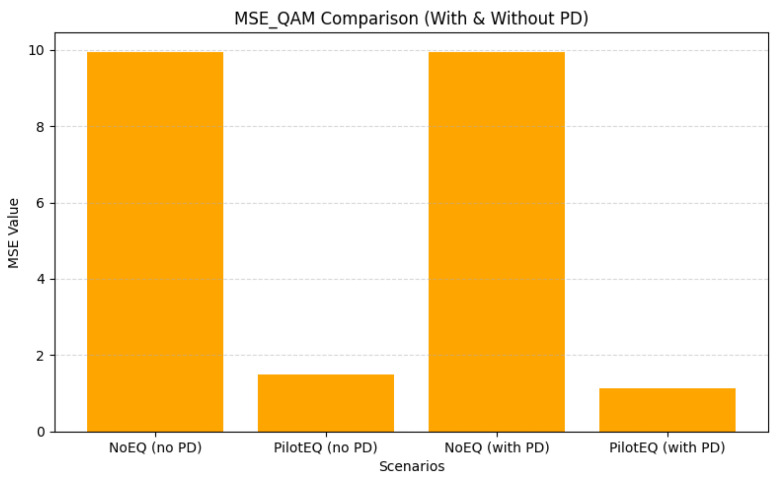
MSE performance for the QAM branch with pilot spacing of 8. Pilot-assisted estimation substantially reduces symbol estimation error relative to the no-pilot case, demonstrating enhanced robustness with and without predistortion.

For a pilot spacing of 8, 16 pilot symbols were inserted within each OFDM frame, resulting in dense sampling of the channel response. Figure 13 shows that pilot-assisted equalization substantially improves performance: the QAM BER drops from 0.267 to 0.048, and Figure 14 shows MSE from 9.954 to 1.133. These gains arise from the improved channel tracking enabled by frequent pilot insertion. As summarized in Table 11, however, this configuration incurs higher overhead and therefore reduces spectral efficiency.

**Figure 15:**
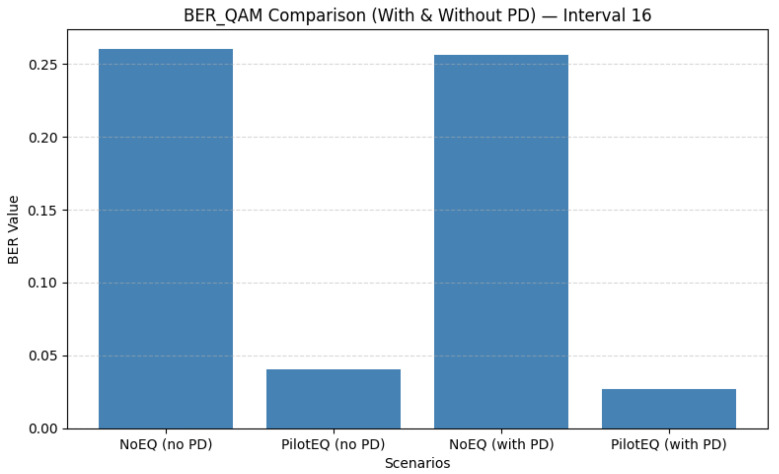
BER performance for the QAM branch with pilot spacing of 16. Dense pilot insertion improves channel estimation accuracy, resulting in significantly lower BER compared to the no-pilot scenario both with and without predistortion.

**Figure 16:**
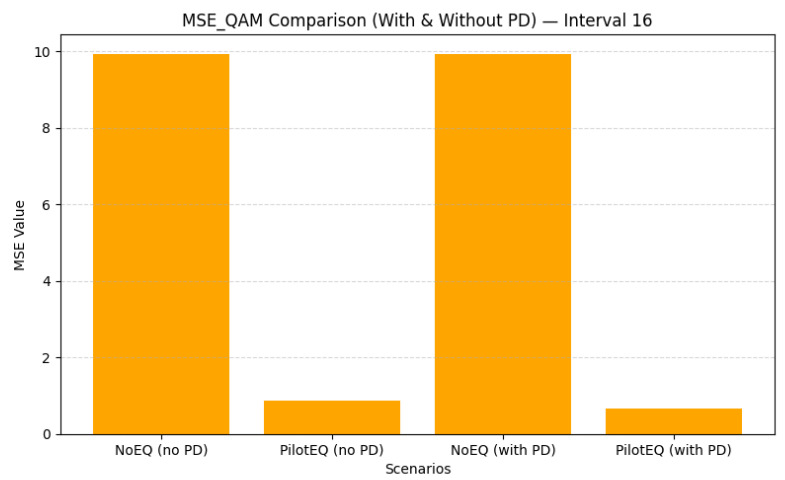
MSE performance for the QAM branch with pilot spacing of 16. Pilot-assisted estimation substantially reduces symbol estimation error relative to the no-pilot case, demonstrating enhanced robustness with and without predistortion.

For a pilot spacing of 16, only eight pilot bins are required, reducing overhead while still providing reliable channel information. As shown in Figure 15, pilot-aided equalization reduces the QAM BER from 0.2606 to 0.0266, and Figure 16 shows MSE from 9.943 to 0.667. These results, detailed in Table 12, indicate that a moderate pilot density can maintain effective channel estimation while improving spectral efficiency compared to the denser spacing of 8.

**Figure 17:**
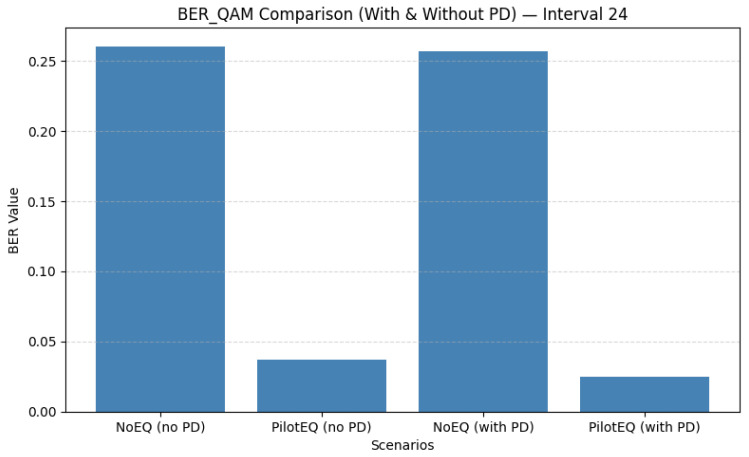
BER performance for the QAM branch with pilot spacing of 24. Dense pilot insertion improves channel estimation accuracy, resulting in significantly lower BER compared to the no-pilot scenario both with and without predistortion.

**Figure 18:**
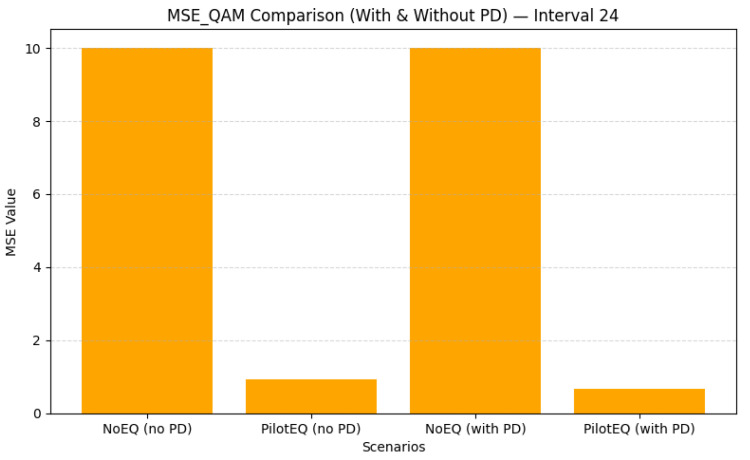
MSE performance for the QAM branch with pilot spacing of 24. Pilot-assisted estimation substantially reduces symbol estimation error relative to the no-pilot case, demonstrating enhanced robustness with and without predistortion.

When the pilot spacing is increased to 24, only six pilot bins per frame are used, offering a significant reduction in overhead while still maintaining reliable interpolation of the channel response. Figure 17 shows that this spacing achieves an excellent balance of performance and efficiency: the QAM BER drops to 0.0248 with PD, and Figure 18 shows that MSE is reduced to 0.667. The corresponding results are summarized in Table 13. Sparse pilot spacing increases throughput, although overly sparse configurations may introduce interpolation errors.

Overall, the comparison across all the tested pilot spacings highlights a clear trade-off between estimation accuracy and spectral efficiency. Dense spacing (8) offers excellent channel tracking but reduces throughput. Intermediate spacing (16) achieves a favorable compromise, while spacing 24 provides the best balance of BER, MSE, and efficiency. Increasing the spacing beyond 24 (e.g., to 25) begins to degrade the MSE due to interpolation limitations, although BER remains low. These results emphasize the importance of carefully selecting pilot density to jointly optimize performance and throughput. Table 14 shows a summary of the QAM BER and MSE for pilot spacings of 8, 16, 24, and 25 subcarriers.

### 3.4. Equalization Approach for PPM Signal

For the PPM signal, a direct time-domain equalization approach was employed. Since PPM concentrates its energy in a single time slot and does not use frequency-multiplexed carriers like QAM, conventional frequency-domain or per-subcarrier equalization methods are not applicable. To establish a baseline, a simple DC-gain equalizer was used and compared against the proposed AI-based equalizer. The BER and MSE with and without PD were measured to assess performance.

Table 15 shows that applying PD reduces the raw PPM BER from 0.4231 to 0.3516 under the strongest nonlinearity condition (α=1.8). Although this numerical reduction reflects a modest improvement in the detectability of the pulse peak, the resulting BER remains far too high for reliable communication and therefore does not constitute usable performance. This outcome confirms that predistortion alone is not sufficient to correct timing distortions induced by severe LED nonlinearity. The MSE remains unchanged at 1.8064, which is expected given that PPM errors are dominated by timing misalignment rather than amplitude scaling. As shown in the next section, only the CNN–TFN equalizer is capable of reducing the PPM BER to practical levels by learning the temporal structure of the distorted pulses.

### 3.5. Deep Learning Model

To further improve equalization, a hybrid convolutional neural network (CNN) and transformer (TFN) architecture was implemented. This design captures both the local nonlinearities and global dependencies across the signal. A batch size of 16 was used for both QAM and PPM models.

The QAM model, which carries information in both amplitude and phase, used a more complex structure: three CNN layers followed by two transformer layers. The PPM model, in contrast, employed one CNN and one transformer layer to avoid overfitting since its simpler structure and sparsity did not require a deep network. The kernel sizes were set to 5 for both models (3 for deeper CNN layers in QAM), and padding was applied to preserve output length.

#### 3.5.1. QAM Analysis

**Figure 19:**
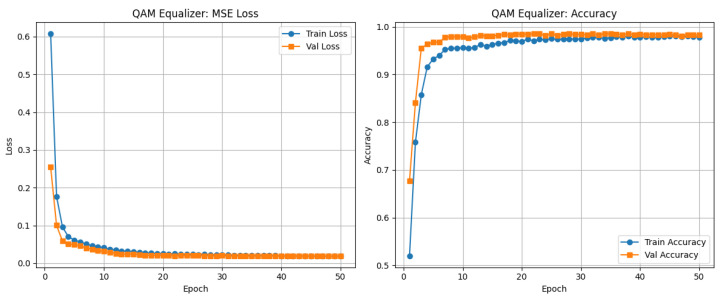
Training and validation loss and accuracy curves for the QAM CNN–TFN equalizer. The model exhibits stable convergence and strong generalization, with validation accuracy approaching 98%.

For the QAM analysis, we constructed a dataset of size (1653,121) for the training and validation of the CNN–TFN equalizer. Each of the 1653 entries corresponds to a decoded OFDM frame, and the 121 elements represent the data-bearing subcarrier amplitudes obtained after FFT demodulation and removal of the pilot, DC, and guard subcarriers. After training, the QAM equalizer achieved a training loss of 0.0239 and a validation loss of 0.0209, with accuracies of 0.9742 and 0.9825, respectively. Figure 19 shows that the validation loss steadily decreased from 0.26 to 0.0209, while the accuracy increased from 0.63 to 0.9825, indicating effective minimization of symbol reconstruction error.

The CNN layers captured local nonlinear distortions, such as LED clipping and inter-carrier interference, while the transformer layers modeled long-range dependencies across subcarriers. The close alignment of the training and validation curves demonstrates good generalization and minimal overfitting. A learning rate of 0.0001 and 50 training epochs were empirically chosen to ensure stable convergence.

**Figure 20:**
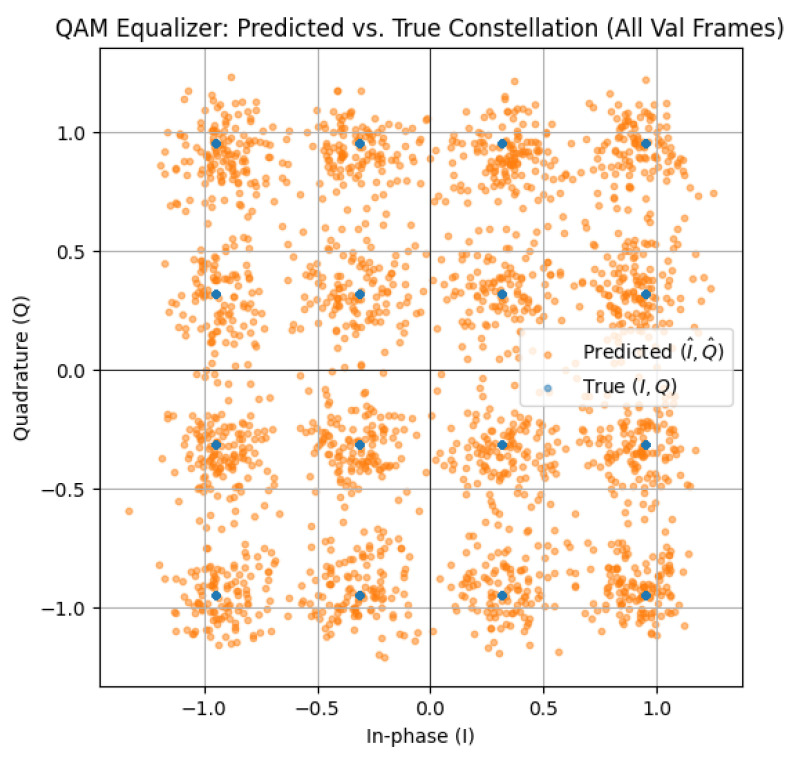
Predicted vs. true 16-QAM constellation points after CNN–TFN equalization. The tight clustering around the ideal symbol positions demonstrates effective compensation of LED and channel distortions.

Figure 20 illustrates the predicted versus true 16-QAM constellation points. Blue points represent the ideal constellation, and orange points represent the model’s predictions across all the validation frames. The predicted symbols cluster tightly around the ideal constellation centroids, showing that the CNN–transformer equalizer effectively compensates for channel distortion. The low spread around each cluster reflects minimal residual error and confirms strong symbol-level reconstruction performance under the tested VLC channel conditions.

#### 3.5.2. PPM Analysis

For the PPM branch, the CNN–TFN equalizer was trained using a dataset of 1653 PPM frames, each consisting of 128 time-domain samples. For performance evaluation, a separate test set was used. Although the original design employed 166 four-PPM symbols (332 bits), the implemented evaluation set contained 331 symbols, corresponding to 662 Gray-coded bits. This larger test set increases the minimum measurable BER resolution compared to Section 3.1.2.

**Figure 21:**
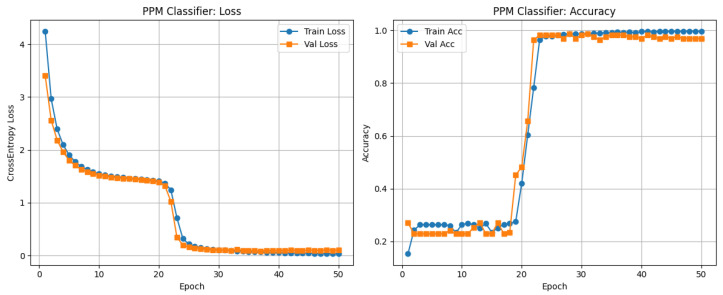
Training and validation loss and accuracy curves for the PPM CNN–TFN equalizer. The model converges rapidly and achieves stable generalization, with accuracy approaching 97%.

The training process, shown in Figure 21, exhibits an initial plateau between epochs 10 and 20, followed by rapid convergence after epoch 22. Both training and validation losses stabilized near zero by epoch 25, with final accuracies of approximately 97%. The close alignment of the training and validation metrics indicates strong generalization and minimal overfitting. Although explicit early stopping was not applied, stabilization around epoch 25 effectively served as an implicit convergence criterion.

In this configuration, the BER was evaluated on the corresponding 662-bit dataset, for which no bit errors were observed; i.e., $\mathrm{BER}<1.51\times 10^{-3}$. Given the finite size of the PPM evaluation set, the reported BER values should be interpreted as upper bounds determined by the test length rather than asymptotic error rates.

**Figure 22:**
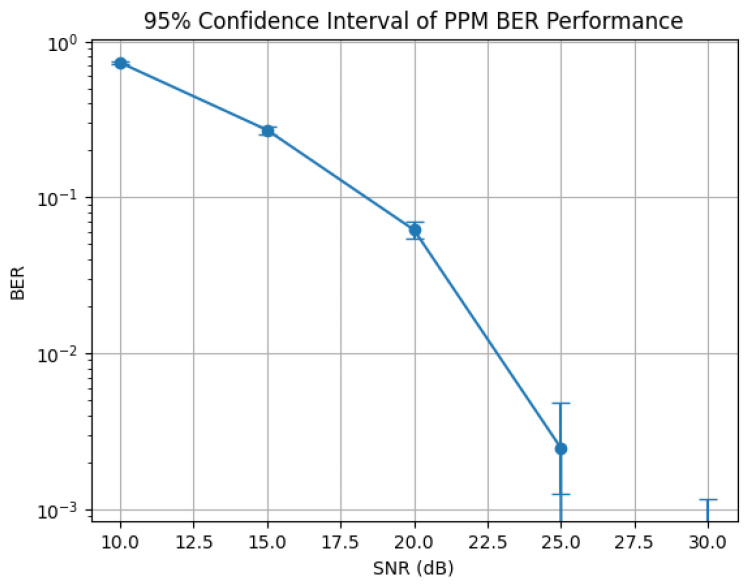
BER performance of the PPM-based VLC system versus SNR with 95% confidence intervals. The BER decreases monotonically with increasing SNR, demonstrating improved detection reliability and near-error-free performance at high SNR.

Figure 22 illustrates the BER performance of the PPM-based VLC system as a function of SNR, including 95% confidence intervals. At low SNR (10 dB), the BER is high due to noise-dominated reception, while increasing the SNR to 15 dB and 20 dB results in a significant BER reduction and improved detection stability, as reflected by narrower confidence intervals. At 25 dB, the BER reaches the order of $10^{-3}$, indicating near-error-free transmission; the slightly wider confidence interval at high SNR is attributed to the reduced number of error events, which increases statistical uncertainty.

### 3.6. Ablation Study

Table 16 summarizes the ablation study conducted to evaluate the contribution of the main system components under identical channel and SNR conditions. Enabling transmitter-side predistortion reduces nonlinear distortion but provides limited performance improvement when used in isolation. Introducing a CNN-based equalizer at the receiver yields a substantial reduction in QAM error rate, confirming the importance of learning-based compensation. The addition of transformer layers further improves performance by capturing long-range dependencies that cannot be effectively modeled by convolutional filters alone. Finally, adaptive bit loading does not directly affect error rate but significantly increases throughput by exploiting subcarrier SNR variations. These results demonstrate that the combined CNN–transformer equalizer with adaptive bit loading provides the most robust and efficient system configuration.

The ablation study highlights the incremental contribution of each system component. Transmitter-side predistortion reduces nonlinear distortion but is insufficient on its own under strong LED nonlinearity. Introducing a CNN-based equalizer at the receiver significantly improves QAM detection accuracy, while the inclusion of transformer layers further enhances performance by capturing long-range dependencies. Finally, adaptive bit loading provides additional throughput gains without degrading error performance. These results confirm that the combined CNN–transformer equalizer and adaptive bit loading yield the most robust and efficient configuration. For the PPM branch, the ablation confirms that learning-based equalization is essential to achieve practical BER levels under strong nonlinearity, while predistortion alone provides only limited improvement.

### 3.7. Analysis Across Different Seeds

**Figure 23:**
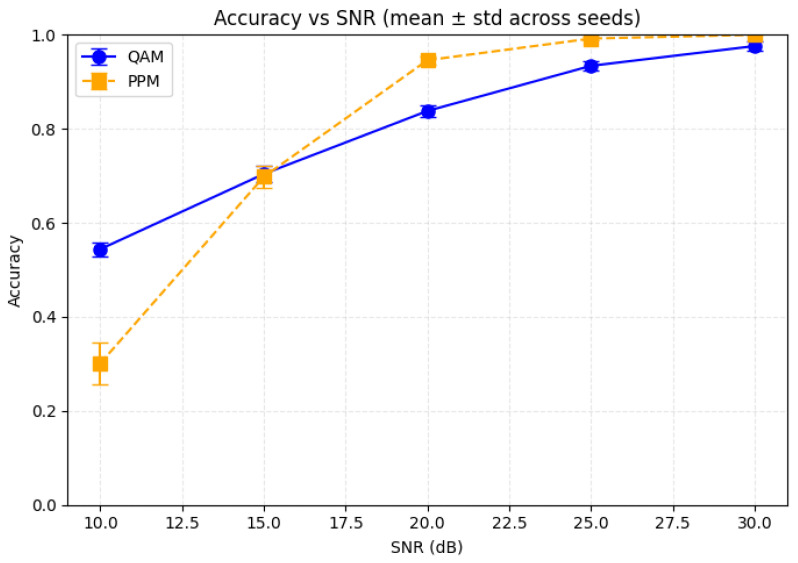
Accuracy of the QAM and PPM CNN–TFN equalizers across different SNR values and random seeds. Both models show stable performance with reduced variability at higher SNRs.

To evaluate robustness, the model was tested using multiple random seeds and various SNR conditions. Figure 23 shows the accuracy trends of both QAM and PPM signals across five seeds for SNR values from 10 to 30 dB, with error bars indicating variability across runs.

QAM exhibits stronger resilience at low and moderate SNRs due to its joint amplitude–phase encoding, which degrades gradually under noise. PPM, in contrast, is more sensitive at low SNRs as weak pulses are easily masked; however, its performance improves rapidly at higher SNRs once pulse positions become distinguishable. As SNR increases, error bars shrink for both modulation schemes, confirming stable convergence and low sensitivity to initialization, indicating that the model architecture is well regularized. Overall, QAM maintains robustness under noisy conditions, while PPM achieves near-perfect accuracy in high-SNR channels.

### 3.8. Adaptive Bit Loading Concept and Throughput Analysis

Adaptive bit loading (ABL) was applied as a post-training simulation step to evaluate how effectively the equalized VLC channel supports varying modulation orders. ABL was performed at a nominal operating SNR of 25 dB. The corresponding noise variance ($\sigma_{w}^{2}=1.11\times 10^{-13}$) was used to compute the instantaneous SNR of each subcarrier, which in turn determined whether 16-QAM or 64-QAM was assigned according to the bit loading threshold rule.

**Figure 24:**
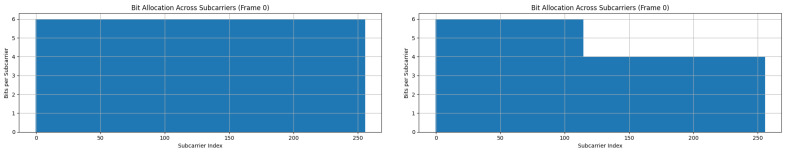
Illustration of uniform bit loading (**left**) and adaptive bit loading (**right**). UBL assigns the same modulation order to all subcarriers, while ABL adapts the bit allocation according to the instantaneous SNR of each subcarrier to improve spectral efficiency.

Figure 24 illustrates uniform bit loading (UBL) versus ABL. Under UBL, all subcarriers use the same modulation order, which is inefficient and may result in information loss under channel variations.

In ABL, bit allocation shifts from 6 to 4 bits per subcarrier as channel quality decreases, e.g., modulating from 64-QAM to 16-QAM. This exploits QAM sensitivity to SNR: assigning higher-order modulation to stronger subcarriers increases spectral efficiency, consistent with OFDM water-filling principles. PPM pulse-position encoding, however, is insensitive to modulation order, so ABL is not applicable.

**Figure 25:**
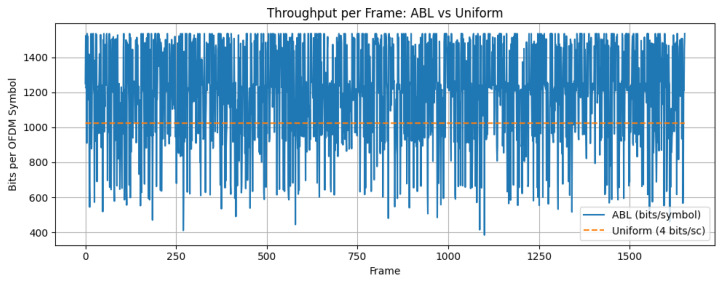
Throughput achieved using adaptive bit loading compared with uniform bit loading. ABL increases average throughput by 16.23% by adapting the modulation order to the instantaneous SNR of each subcarrier.

Figure 25 shows the throughput comparison. The ABL configuration achieved an average throughput of 1190.15 bits/OFDM symbol, a 16.23% improvement over uniform 16-QAM (1024 bits/symbol). The observed standard deviation (279.1 bits/symbol) of the throughput distribution obtained over many OFDM frames reflects the natural throughput fluctuations introduced by adaptive bit loading as the modulation order assigned to each subcarrier changes according to its instantaneous SNR. These results confirm that ABL significantly enhances spectral efficiency and system capacity in moderate-SNR VLC channels.

## 4. Discussion

This section discusses the implications of the numerical results obtained for the proposed hybrid VLC–OFDM system, focusing on the CNN–transformer equalizer, the neural and polynomial predistorters (PDs), and the adaptive bit loading scheme.

The CNN–TFN equalizer demonstrates stable learning dynamics and strong generalization across SNR levels for both the QAM and PPM branches. For QAM, the training and validation curves in Figure 19 converge smoothly, and the constellation plot in Figure 20 shows tight clustering of the predicted symbols around the ideal 16-QAM points. This indicates that the model is able to compensate for nonlinear LED distortions and channel attenuation without overfitting. For PPM, the reduced-depth CNN–TFN architecture achieves high classification accuracy while limiting model complexity: training and validation accuracy reach approximately 97% in Figure 21, confirming that the network effectively captures temporal pulse-position information under nonlinear and noisy conditions.

The use of convolutional layers at the front-end allows the model to extract local features associated with nonlinear distortion and residual inter-symbol interference, whereas the transformer layers capture longer-range dependencies across samples and subcarriers. This division of roles is consistent with the observed improvements in symbol reconstruction quality and the compact constellation distributions in Figure 20. For PPM, the shallower architecture avoids over-parameterization while still providing sufficient capacity to learn timing patterns in the received waveform.

In addition to receiver-side equalization, a CNN-based PD was investigated as an alternative to a fixed polynomial PD. Although the absolute BER improvements over the simple PD are modest (e.g., QAM BER reduction from 0.0483 to 0.0475 in Table 9), they are consistent across epochs and demonstrate that a data-driven PD can capture subtle nonlinear effects that are not fully modeled by a static polynomial. The PPM branch exhibits much smaller variations, in line with its intrinsic robustness to amplitude distortions and its primary sensitivity to timing errors.

Adaptive bit loading further enhances the overall system efficiency by tailoring the modulation order to the instantaneous SNR of each subcarrier. As shown in Figure 25, ABL increases the average throughput from 1024 bits/OFDM symbol (uniform 16-QAM) to 1190.15 bits/OFDM symbol, corresponding to a 16.23% gain, while maintaining low BER. The variability in the achieved throughput (279.1 bits/symbol) reflects the fact that bit allocation is channel-dependent: when more subcarriers exceed the 64-QAM threshold, the instantaneous throughput increases and vice versa. This behavior is consistent with the goal of exploiting frequency selectivity and unequal subcarrier SNRs to improve spectral efficiency.

The robustness of the proposed equalizer to randomness in initialization and to SNR variations is confirmed in Figure 23 and Table 17. Across multiple random seeds and SNR values, QAM accuracy increases steadily with SNR, while PPM rapidly approaches near-perfect accuracy at moderate-to-high SNR. The spread across seeds remains limited, indicating that the CNN–TFN model does not rely on a specific initialization and is resilient to channel noise realizations.

Compared with classical equalization strategies and single-architecture deep models (e.g., purely CNN- or LSTM-based approaches reported in the literature), the proposed CNN–TFN design combines local feature extraction with global attention, which is particularly suitable for OFDM-based VLC links affected by both nonlinear distortion and frequency selectivity. While a detailed head-to-head benchmark with alternative deep equalizers is beyond the scope of this work, the reported BER, MSE, and accuracy metrics demonstrate that the hybrid architecture is competitive and well matched to the considered channel and hardware model. The joint use of CNN–TFN equalization and ABL, which, to the best of our knowledge, has not been widely explored for hybrid QAM–PPM VLC systems, leads to simultaneous improvements in reliability and throughput.

This study is limited to simulation-based evaluation with a modeled LED nonlinearity, idealized synchronization, and a quasi-static channel. Nevertheless, the quantitative gains observed in BER, MSE, and throughput indicate that combining CNN–TFN equalization with ABL and neural predistortion is a promising strategy for practical VLC–OFDM systems. Future work will include experimental validation with real hardware, investigation of mobility and pointing errors in body area network scenarios, and the integration of channel coding to translate the observed symbol-level improvements into end-to-end link-level gains. In particular, the adopted memoryless channel model does not capture frequency-selective dispersion effects that may arise in larger indoor environments or highly reflective scenarios; extending the proposed framework to dispersive VLC channels is an important direction for future work. In this work, the VLC channel is modeled as memoryless in the frequency domain, which is a common assumption in OFDM-based VLC systems. Although indoor and BAN environments may introduce temporal dispersion due to reflections and scattering, the proposed system employs OFDM with a cyclic prefix and guard interval. The cyclic prefix length is chosen to exceed the expected channel delay spread, thereby mitigating inter-symbol interference and enabling each subcarrier to experience a flat-fading memoryless channel. As a result, the memoryless channel assumption is valid at the subcarrier level for the considered system model.

While the proposed CNN–transformer equalizer introduces higher computational complexity than classical linear equalizers, the performance gains in BER and spectral efficiency are achieved with a relatively shallow architecture. The separation between QAM and PPM processing and the use of a lightweight model for the PPM branch further reduce inference cost. These characteristics suggest that the proposed approach is feasible for BAN-oriented VLC receivers where moderate processing capability is available, while more constrained nodes could adopt simplified architectures as a trade-off between complexity and performance.

From a computational perspective, the proposed CNN–transformer equalizer introduces higher complexity than classical linear equalizers or polynomial predistortion alone due to the use of convolutional layers and self-attention mechanisms. In this work, complexity is discussed in relative terms based on architectural depth, number of layers, and dominant operations rather than absolute runtime or hardware-specific metrics. The architecture is intentionally kept shallow, with a limited number of convolutional and transformer encoder blocks, and a reduced-depth model is adopted for the PPM branch to limit inference cost. Compared to CNN-only equalization, the inclusion of transformer layers increases computational overhead but yields improved robustness and accuracy, representing a deliberate trade-off between performance and complexity. A detailed hardware-level complexity or real-time efficiency analysis is beyond the scope of this simulation-based study.

A direct quantitative benchmark against all the existing learning-based VLC receivers is beyond the scope of this work. Nevertheless, it is instructive to qualitatively compare the proposed CNN–transformer equalizer with representative approaches reported in the literature. CNN-only receivers have been shown to effectively compensate local nonlinear distortions, but their limited receptive field can hinder modeling of long-range dependencies, particularly in OFDM-based systems. Recurrent architectures, such as LSTM-based equalizers, can capture temporal correlations but often incur higher training complexity and latency. In contrast, the proposed CNN–transformer architecture combines local feature extraction with global self-attention, enabling effective modeling of both nonlinear distortion and long-range dependencies within a unified framework. Furthermore, most existing works focus on single-modulation VLC schemes, whereas the proposed approach explicitly targets a hybrid QAM–PPM system with adaptive bit loading, which is not commonly addressed in prior studies.

## 5. Conclusions and Future Work

This paper proposed an AI-enhanced hybrid VLC–OFDM framework that combines QAM and PPM modulation with deep learning-based equalization and adaptive bit loading, targeting body area network scenarios affected by LED nonlinearity and channel impairments. A CNN–transformer equalizer was developed to jointly process the two modulation branches, while adaptive bit loading dynamically adjusted the modulation order of the QAM branch based on subcarrier SNR. The system was evaluated through an end-to-end simulation framework under realistic noise and nonlinearity conditions.

The results demonstrate that the proposed CNN–TFN equalizer significantly improves symbol reconstruction accuracy for both modulation schemes. For QAM, effective compensation of nonlinear distortion leads to consistently low BER, while, for PPM, a reduced-complexity architecture achieves high classification accuracy without overfitting. The analysis of predistortion strategies shows that a fifth-order polynomial PD provides the best balance between performance and complexity. In addition, adaptive bit loading increases the average throughput by more than 16% compared to uniform 16-QAM, confirming the benefit of combining AI-based equalization with adaptive transmission strategies.

Future work will focus on experimental validation using real LED–photodiode front-ends, enabling the assessment of mobility, device-specific nonlinearities, and ambient interference in practical BAN environments. Extensions toward dispersive channel models, online or real-time learning, and additional adaptive mechanisms such as power allocation or beamforming represent promising directions to further enhance the robustness and applicability of VLC systems.

## Figures and Tables

**Table 1:** Optical channel gains for LOS, NLOS, and total paths.

Component	Gain
$H_{\mathrm{LOS}}$	$7.9577\times 10^{-5}$
$H_{\mathrm{NLOS}}$	$1.7506\times 10^{-6}$
$H_{\mathrm{Total}}$	$9.7084\times 10^{-5}$
Signal power	$6.32\times 10^{-12}$
Noise power	$9.72\times 10^{-15}$
SNR (dB)	26.13

**Table 2:** System and channel parameters used in the VLC simulation model, including LED characteristics, receiver configuration, physical environment settings, and noise-related values.

Parameter	Value	Rationale
Distance	2.0 m	Represents typical indoor LOS scenarios
Lambertian order	1.0	Moderately wide LED emission pattern for realistic coverage
Photodetector area ($A_{\mathrm{PD}}$)	$1\times 10^{-4}\;m^{2}$	Typical area for indoor VLC photodetector
Filter gain	1.0	Neutral filter for simplicity
Concentrator gain	1.0	Unity gain to isolate other effects
Reflectivity	0.7	Typical indoor wall/ceiling reflectivity
Reflector distance	[3.0 m, 4.0 m]	Models multipath reflection distance
Reflector angle	[45°, 60°]	Reflects common indoor surface angles
SNR	30 dB	High-quality link while allowing measurable degradation
Noise bandwidth (*B*)	$10\times 10^{6}$ Hz	Represents typical photodetector bandwidth
Temperature (*T*)	300 K	Standard room temperature
Load resistance ($R_{\mathrm{load}}$)	50 Ω	Common in electronics for impedance matching
Ambient irradiance ($E_{\mathrm{amb}}$)	$0.01\;{W/m}^{2}$	Simulates low-level ambient light interference
Responsivity (*r*)	0.7 A/W	Typical photodetector responsivity

**Table 3:** Transformer–CNN hybrid model architecture.

Layer Type	Output Shape	Params	Remarks
Conv1d	(16, 64, 119)	704	First convolution for initial feature extraction
ReLU	(16, 64, 119)	0	Nonlinearity
Conv1d	(16, 128, 119)	24,704	Deeper feature representation
ReLU	(16, 128, 119)	0	Activation
Conv1d	(16, 128, 119)	258	Convolution with same channels
Dropout	(16, 128, 119)	0	Regularization
PositionalEncoding	(16, 128, 119)	0	Adds temporal context
LayerNorm	(16, 119, 128)	256	Normalization before attention
MultiheadAttention	(–1, 119, 128) → (–1, 119, 128)	0	Self-attention layer
Dropout	(16, 119, 128)	0	Regularization after attention
LayerNorm	(16, 119, 128)	256	Stabilizes FFN input
Linear	(16, 119, 256)	33,024	Expands dimensionality
ReLU	(16, 119, 256)	0	Nonlinearity
Dropout	(16, 119, 256)	0	Prevents overfitting
Linear	(16, 119, 128)	32,896	Compresses feature back
Dropout	(16, 119, 128)	0	Further regularization
TransformerEncoderBlock	(16, 119, 128)	0	End of 1st transformer encoder block
LayerNorm	(16, 119, 128)	256	Normalization for 2nd encoder
MultiheadAttention	(–1, 119, 128) → (–1, 119, 128)	0	Self-attention layer
Dropout	(16, 119, 128)	0	Regularization
LayerNorm	(16, 119, 128)	256	Normalization
Linear	(16, 119, 256)	33,024	FFN expansion
ReLU	(16, 119, 256)	0	Nonlinearity
Dropout	(16, 119, 256)	0	Prevents overfitting
Linear	(16, 119, 128)	32,896	Reduces dimensionality
Dropout	(16, 119, 128)	0	Dropout
TransformerEncoderBlock	(16, 119, 128)	0	End of 2nd transformer encoder block
Linear	(16, 119, 2)	258	Maps to symbol space
Linear	(16, 119, 2)	6	Final projection for output

**Table 4:** Model architecture summary for PPM classifier.

Layer (Type)	Output Shape	Params	Remarks
Conv1D-1	[16, 64, 128]	384	Initial convolution for local temporal feature extraction.
ReLU-2	[16, 64, 128]	0	Nonlinear activation improving feature separability.
PositionalEncoding-3	[16, 128, 64]	0	Adds temporal-order information via sinusoidal encoding.
MultiHeadAttention-4	[16, 128, 64]	0	Captures long-range temporal dependencies.
Dropout-5	[16, 128, 64]	0	Regularization to reduce overfitting.
LayerNorm-6	[16, 128, 64]	128	Normalizes activations before FFN for stability.
Linear-7	[16, 128, 128]	8320	FFN expansion increasing representation capacity.
ReLU-8	[16, 128, 128]	0	Nonlinearity inside the feed-forward network.
Linear-9	[16, 128, 64]	8256	FFN compression projecting features back to 64 dims.
Dropout-10	[16, 128, 64]	0	Regularization after FFN to prevent overfitting.
LayerNorm-11	[16, 128, 64]	128	Layer normalization before the next encoder block.
TransformerEncoderBlock-12	[16, 128, 64]	0	Second encoder block refining temporal representations.
GlobalAvgPool-13	[16, 64]	0	Pools temporal features into a global embedding.
Linear-14 (Output)	[16, 128]	8320	Final classifier projecting features to PPM symbol logits.

**Table 5:** BER and MSE comparison for QAM and PPM signals with and without PD. Case α=1.0 and 3rd-order PD.

Scenario	${\mathrm{BER}}_{\mathrm{QAM}}$	${\mathrm{BER}}_{\mathrm{PPM}}$	${\mathrm{MSE}}_{\mathrm{QAM}}$	${\mathrm{MSE}}_{\mathrm{PPM}}$
Without PD	0.0962	0	2.269	2.811
With PD	0.0800	0	1.909	2.811

**Table 6:** BER and MSE comparison for QAM and PPM signals with 5th-order PD and α=0.5, showing improvements due to predistortion.

Scenario	${\mathrm{BER}}_{\mathrm{QAM}}$	${\mathrm{BER}}_{\mathrm{PPM}}$	${\mathrm{MSE}}_{\mathrm{QAM}}$	${\mathrm{MSE}}_{\mathrm{PPM}}$
Without PD	0.0629	<0.00602	1.395	2.811
With PD	0.0456	<0.00602	1.051	2.811

**Table 7:** BER and MSE comparison for 7th-order PD and α=1.8. Higher-order PD improves performance for both QAM and PPM.

Scenario	${\mathrm{BER}}_{\mathrm{QAM}}$	${\mathrm{BER}}_{\mathrm{PPM}}$	${\mathrm{MSE}}_{\mathrm{QAM}}$	${\mathrm{MSE}}_{\mathrm{PPM}}$
Without PD	0.0625	0.4230	0.1306	1.806
With PD	0.0472	0.3406	0.1350	1.806

**Table 8:** BER and MSE performance of the hybrid VLC system using the conventional polynomial predistorter (simple PD).

Scenario	${\mathrm{BER}}_{\mathrm{QAM}}$	${\mathrm{BER}}_{\mathrm{PPM}}$	${\mathrm{MSE}}_{\mathrm{QAM}}$	${\mathrm{MSE}}_{\mathrm{PPM}}$
With Simple PD	0.0483	0.3516	1.138	1.806

**Table 9:** Evolution of BER and MSE for QAM and PPM across training epochs of the CNN-based predistorter. Results show the convergence behavior and the modest performance gains achieved by the neural PD.

Epoch	${\mathrm{BER}}_{\mathrm{QAM}}$	${\mathrm{BER}}_{\mathrm{PPM}}$	${\mathrm{MSE}}_{\mathrm{QAM}}$	${\mathrm{MSE}}_{\mathrm{PPM}}$
10	0.0524	0.3417	1.163	1.806
20	0.0495	0.3406	1.163	1.806
30	0.0489	0.3406	1.160	1.806
40	0.0508	0.3406	1.155	1.806
50	0.0502	0.3406	1.152	1.806
60	0.0487	0.3510	1.135	1.806
70	0.0483	0.3570	1.132	1.806
80	0.0480	0.3510	1.133	1.806
90	0.0493	0.3400	1.157	1.806
100	0.0475	0.3500	1.137	1.806

**Table 10:** BER and MSE values for QAM and PPM under different PD orders at α=1.8. The results show that increasing the PD order significantly improves QAM accuracy, while PPM exhibits only minor changes due to its robustness to amplitude distortion.

PD Order	${\mathrm{BER}}_{\mathrm{QAM}}$	${\mathrm{BER}}_{\mathrm{PPM}}$	${\mathrm{MSE}}_{\mathrm{QAM}}$	${\mathrm{MSE}}_{\mathrm{PPM}}$
1st	0.0643	0.357	1.4520	1.806
3rd	0.0524	0.357	1.2030	1.806
5th	0.0483	0.352	1.2380	1.806
7th	0.0472	0.345	1.1200	1.806

**Table 11:** BER and MSE comparison for the QAM branch under a pilot spacing of 8. Pilot-assisted equalization greatly improves accuracy but introduces notable overhead, reducing spectral efficiency.

Method	${\mathrm{BER}}_{\mathrm{QAM}}$	${\mathrm{MSE}}_{\mathrm{QAM}}$
NoEQ (no PD)	0.267	9.954
PilotEQ (no PD)	0.067	1.492
NoEQ (with PD)	0.262	9.956
PilotEQ (with PD)	0.048	1.133

**Table 12:** BER and MSE comparison for the QAM branch under a pilot spacing of 16. Results show that moderate pilot density maintains effective equalization with less overhead than dense pilot insertion.

Method	${\mathrm{BER}}_{\mathrm{QAM}}$	${\mathrm{MSE}}_{\mathrm{QAM}}$
NoEQ (no PD)	0.2606	9.9431
PilotEQ (no PD)	0.0406	0.871
NoEQ (with PD)	0.256	9.943
PilotEQ (with PD)	0.0266	0.667

**Table 13:** BER and MSE comparison for the QAM branch under a pilot spacing of 24. Sparse pilot insertion improves throughput while maintaining accurate equalization and low BER.

Method	${\mathrm{BER}}_{\mathrm{QAM}}$	${\mathrm{MSE}}_{\mathrm{QAM}}$
NoEQ (no PD)	0.2606	10.0143
PilotEQ (no PD)	0.037	0.931
NoEQ (with PD)	0.257	10.0143
PilotEQ (with PD)	0.0248	0.667

**Table 14:** Summary of QAM BER and MSE for pilot spacings of 8, 16, 24, and 25 subcarriers. The optimal performance–efficiency balance is obtained at a spacing of 24, while larger spacings (e.g., 25) begin to introduce interpolation errors.

Pilot Spacing	BER_QAM_	MSE_QAM_
8	0.048	1.13
16	0.0266	0.667
24	0.0248	0.693
25	0.0264	0.693

**Table 15:** BER and MSE of the PPM branch with and without PD under strong LED nonlinearity (α=1.8). PD improves pulse-detection accuracy, reducing BER, while MSE remains unchanged due to the amplitude-insensitive nature of PPM.

Method	BER_PPM_	MSE_PPM_
Without PD	0.4231	1.8064
With PD	0.3516	1.8064

**Table 16:** Ablation study evaluating the contributions of key system components under identical channel and SNR conditions.

Configuration	PD	CNN	Transformer	ABL	BER_QAM_	Throughput Gain
Baseline (no AI, no PD)	×	×	×	×	High	–
PD only	✓	×	×	×	↓	–
CNN equalizer (no TFN)	✓	✓	×	×	↓↓	–
CNN–TFN equalizer	✓	✓	✓	×	↓↓↓	–
CNN–TFN + ABL (full system)	✓	✓	✓	✓	Lowest	+16%

**Table 17:** Accuracy of the QAM and PPM CNN–TFN equalizers across different SNR values and random seeds. Results show consistent performance trends and reduced variability at higher SNR.

Case	Modulation	10 dB	15 dB	20 dB	25 dB	30 dB
1	QAM	0.55	0.72	0.85	0.94	0.98
2	QAM	0.54	0.70	0.84	0.94	0.98
3	QAM	0.55	0.70	0.83	0.93	0.98
4	QAM	0.52	0.68	0.82	0.92	0.96
5	QAM	0.56	0.72	0.85	0.94	0.98
1	PPM	0.275	0.68	0.94	0.99	1.00
2	PPM	0.27	0.70	0.94	0.99	1.00
3	PPM	0.37	0.71	0.95	0.99	1.00
4	PPM	0.32	0.73	0.96	0.99	1.00
5	PPM	0.27	0.67	0.94	1.00	1.00

## Data Availability

The raw data supporting the conclusions of this article will be made available by the authors on request.

## Appendix A. VLC Channel and Noise Modeling

The deterministic LOS component is modeled following the standard DC gain expression for intensity-modulated direct-detection VLC links:

(A1) $$H_{\mathrm{LOS}}=\frac{(\ell +1)A}{2\pi d^{2}}\cos^{\ell }\left(\phi \right)\;T_{s}\left(\psi \right)\;g\left(\psi \right)\;\cos\left(\psi \right),$$

where *ℓ* is the Lambertian emission order of the LED, *A* is the photodetector area, *d* is the transmitter–receiver separation, ϕ is the irradiance angle, and ψ is the incidence angle. $T_{s}\left(\psi \right)$ denotes the optical filter gain at incidence angle ψ. It models the transmission of any optical filter placed in front of the photodiode. In systems without optical filtering, $T_{s}\left(\psi \right)=1$ for all $\psi \leq \Psi_{c}$, where $\Psi_{c}$ is the receiver field of view (FOV). The term g(ψ) denotes the concentrator gain,

(A2) $$g\left(\psi \right)=\left\{\begin{array}{cc} \frac{n_{c}^{2}}{\sin^{2}\left(\Psi_{c}\right)}, & 0\leq \psi \leq \Psi_{c},\\ 0, & \psi >\Psi_{c}, \end{array}\right.$$

where $n_{c}$ is the refractive index of the optical concentrator and $\Psi_{c}$ the receiver FOV. A Lambertian order ℓ=1 and a distance d=2 m are used to represent a typical downlink from an overhead LED to a wearable receiver located on the user’s body.

In addition to the direct LOS component, reflections from surrounding surfaces contribute a first-order non-line-of-sight (NLOS) optical gain. Following the standard Lambertian reflection model, the NLOS DC gain is obtained by integrating the contribution of each differential surface element dA:

(A3) $$H_{\mathrm{NLOS}}=\underset{A_{\mathrm{ref}}}{\;\int \int \;}\frac{(\ell +1)\;\rho }{2\pi^{2}}\;\frac{A_{\mathrm{PD}}}{d_{1}^{2}d_{2}^{2}}\;\cos^{\ell }\left(\phi_{1}\right)\;\cos\left(\phi_{2}\right)\;\cos\left(\psi \right)\;T_{s}\left(\psi \right)\;g\left(\psi \right)\;dA,$$

where $A_{\mathrm{ref}}$ is the reflecting surface, ρ is its reflectance, $d_{1}$ is the distance from the LED to the surface element, $d_{2}$ is the distance from the surface element to the receiver, $\phi_{1}$ is the irradiance angle at the LED, $\phi_{2}$ is the angle between the surface normal and the reflected ray, and ψ is the incidence angle at the receiver. The functions $T_{s}\left(\psi \right)$ and g(ψ) are defined in (A2).

For numerical evaluation, the reflecting surfaces may be discretized into *K* small patches of area $\Delta A_{k}$, yielding the approximation

(A4) $$H_{\mathrm{NLOS}}\approx \sum_{k=1}^{K}\frac{(\ell +1)\;\rho }{2\pi^{2}}\;\frac{A_{\mathrm{PD}}}{d_{1,k}^{2}d_{2,k}^{2}}\;\cos^{\ell }\left(\phi_{1,k}\right)\;\cos\left(\phi_{2,k}\right)\;\cos\left(\psi_{k}\right)\;T_{s}\left(\psi_{k}\right)\;g\left(\psi_{k}\right)\;\Delta A_{k}.$$

Diffuse reflection coefficient ρ and geometric parameters (reflector distance and angle) are listed in Table 2. The NLOS contribution $H_{\mathrm{NLOS}}$ is computed by integrating the reflected irradiance over the visible reflective surface.

The received electrical signal follows the discrete-time scalar channel model introduced in (6). Specifically, y[n] denotes the received electrical sample at time index *n*, corresponding to the optical signal emitted by the LED after propagation through the channel. The term w[n] represents the additive noise affecting the received signal. The noise sequence w[n] is modeled as an independent and identically distributed (i.i.d.) Gaussian (normal) random process with zero mean and variance $\sigma_{w}^{2}$, denoted as

(A5) $$w\left[n\right]∼N\left(0,\sigma_{w}^{2}\right),$$

where N(·) denotes the Gaussian distribution. This model captures the combined effects of thermal noise in the receiver circuitry, shot noise arising from the photodetection process, and disturbances induced by ambient light. The noise variance is calculated using standard photodetector noise models based on the parameters in Table 2, including noise bandwidth *B*, temperature *T*, load resistance $R_{\mathrm{load}}$, ambient irradiance $E_{\mathrm{amb}}$, and photodiode responsivity *r*.

The electrical signal-to-noise ratio (SNR) at the receiver is defined as

(A6) $$\mathrm{SNR}=\frac{H_{\mathrm{Total}}^{2}\;E\left[y^{2}\left[n\right]\right]}{\sigma_{w}^{2}}.$$

A nominal operating point of approximately 30 dB is used in the simulation study to represent a high-quality BAN-oriented VLC link that still experiences observable nonlinear and noise-induced degradation. This allows meaningful comparison of different predistortion orders, equalization strategies, and adaptive bit loading configurations.

## Appendix B. Detailed Predistortion Performance Results

Table A1 reports the detailed BER and MSE values obtained for different polynomial predistorter orders and LED nonlinearity coefficients. These results are provided for completeness and support the performance trends discussed in Section 3.

**Table A1:** Summary of BER and MSE for various PD orders, nonlinearity levels, and PD activation. Higher-order PD consistently improves QAM and increasingly benefits PPM at high α.

α	Order	PD	BER_QAM_	MSE_QAM_	BER_PPM_	MSE_PPM_
0.5	1st	On	0.0610	1.3890	<0.00602	2.811
	1st	Off	0.0629	1.3950	<0.00602	2.811
	3rd	On	0.0476	1.1030	<0.00602	2.811
	3rd	Off	0.0629	1.3930	<0.00602	2.811
	5th	On	0.0456	1.8509	<0.00602	2.811
	5th	Off	0.0629	1.3950	<0.00602	2.811
1.0	1st	On	0.0930	2.1390	<0.00602	2.811
	1st	Off	0.0961	2.2690	<0.00602	2.811
	3rd	On	0.0800	1.9090	<0.00602	2.811
	3rd	Off	0.0961	2.2600	<0.00602	2.811
	5th	On	0.0747	1.7790	<0.00602	2.811
	5th	Off	0.0961	2.2690	<0.00602	2.811
1.8	1st	On	0.0643	1.4520	0.357	1.806
	1st	Off	0.0658	1.4930	0.423	1.806
	3rd	On	0.0524	1.2030	0.357	1.806
	3rd	Off	0.0658	1.4920	0.423	1.806
	5th	On	0.0483	1.2380	0.352	1.806
	5th	Off	0.0658	1.4920	0.423	1.806
	7th	On	0.0472	1.1200	0.345	1.800
	7th	Off	0.0658	1.4920	0.423	1.806
